# Unmasking selective path integration deficits in Alzheimer’s disease risk carriers

**DOI:** 10.1126/sciadv.aba1394

**Published:** 2020-08-28

**Authors:** Anne Bierbrauer, Lukas Kunz, Carlos A. Gomes, Maike Luhmann, Lorena Deuker, Stephan Getzmann, Edmund Wascher, Patrick D. Gajewski, Jan G. Hengstler, Marina Fernandez-Alvarez, Mercedes Atienza, Davide M. Cammisuli, Francesco Bonatti, Carlo Pruneti, Antonio Percesepe, Youssef Bellaali, Bernard Hanseeuw, Bryan A. Strange, Jose L. Cantero, Nikolai Axmacher

**Affiliations:** 1 Department of Neuropsychology, Institute of Cognitive Neuroscience, Faculty of Psychology, Ruhr University Bochum, Universitätsstraße 150, 44801 Bochum, Germany.; 2 Epilepsy Center, Medical Center–University of Freiburg, Faculty of Medicine, University of Freiburg, Breisacher Str. 64, 79106 Freiburg im Breisgau, Germany.; 3 Spemann Graduate School of Biology and Medicine (SGBM), University of Freiburg, Freiburg, Germany.; 4 Faculty of Biology, University of Freiburg, Schänzlestraße 1, 79104 Freiburg, Germany.; 5 Faculty of Psychology, Ruhr University Bochum, Bochum, Germany.; 6 Leibniz Research Centre for Working Environment and Human Factors (IfADo), Technical University of Dortmund, Dortmund, Germany.; 7 Laboratory of Functional Neuroscience, Pablo de Olavide University, Network Center for Biomedical Research in Neurodegenerative Disease (CIBERNED), Seville, Spain.; 8 Department of Medicine and Surgery, Laboratory of Clinical Psychology, Clinical Psychophysiology and Clinical Neuropsychology, University of Parma, Parma, Italy.; 9 Department of Medicine and Surgery, Medical Genetics, University of Parma, Parma, Italy.; 10 Department of Neurology, Cliniques Universitaires Saint-Luc, Institute of Neuroscience, Université Catholique de Louvain, Brussels, Belgium.; 11 Gordon Center for Medical Imaging, Department of Radiology, Massachusetts General Hospital, Harvard Medical School, Boston, MA, USA.; 12 Department of Neuroimaging, Alzheimer’s Disease Research Centre, Reina Sofia–CIEN Foundation, Madrid, Spain.; 13 Laboratory for Clinical Neuroscience, Centre for Biomedical Technology, Universidad Politecnica de Madrid, Madrid, Spain.; 14 State Key Laboratory of Cognitive Neuroscience and Learning and IDG/McGovern Institute for Brain Research, Beijing Normal University, Xinjiekouwai Street 19, Beijing 100875, China.

## Abstract

Alzheimer’s disease (AD) manifests with progressive memory loss and spatial disorientation. Neuropathological studies suggest early AD pathology in the entorhinal cortex (EC) of young adults at genetic risk for AD (*APOE* ε4-carriers). Because the EC harbors grid cells, a likely neural substrate of path integration (PI), we examined PI performance in *APOE* ε4-carriers during a virtual navigation task. We report a selective impairment in *APOE* ε4-carriers specifically when recruitment of compensatory navigational strategies via supportive spatial cues was disabled. A separate fMRI study revealed that PI performance was associated with the strength of entorhinal grid-like representations when no compensatory strategies were available, suggesting grid cell dysfunction as a mechanistic explanation for PI deficits in *APOE* ε4-carriers. Furthermore, posterior cingulate/retrosplenial cortex was involved in the recruitment of compensatory navigational strategies via supportive spatial cues. Our results provide evidence for selective PI deficits in AD risk carriers, decades before potential disease onset.

## Teaser

Alzheimer’s risk carriers exhibit impaired path integration performance when compensatory strategies cannot be used.

## INTRODUCTION

Alzheimer’s disease (AD), by far the most common form of dementia, is characterized by a progressive deterioration of cognitive functions, starting with episodic memory loss and spatial disorientation (*1*). No causal therapies for AD are currently available, possibly because drugs that would otherwise be effective are applied too late (*2*). Hence, developing biomarkers and behavioral tests for identifying subjects at risk for developing AD is a crucial goal of current AD research.

The ε4 allele of the apolipoprotein E (*APOE*) gene is the most important genetic risk factor for late-onset AD (*3*). Thus, it may provide an opportunity for assessing subclinical alterations of behavior, brain structure, and brain function at very early disease stages (*1*, *4*). However, previous studies and meta-analyses on *APOE*-behavior relationships in young and middle-aged healthy participants showed divergent results: While some described cognitive impairments [e.g., (*5*)], others reported improved functioning [e.g., (*6*)]—suggesting antagonistic pleiotropy—or no effects [e.g., (*4*, *5*, *7*)].

Here, we hypothesized that these divergent findings occur because *APOE* ε4 effects on behavior are mediated by subtle, preclinical AD pathology in confined brain regions, which may be compensated by increased recruitment of unaffected areas—possibly resulting in altered cognitive strategies. Postmortem brain studies revealed first signs of neurodegeneration in the form of neurofibrillary tangles in *APOE* ε4-carriers already in early adulthood (*8*). Because tau-related neurodegeneration correlates closely with cognitive dysfunction (*9*), this very early tauopathy may manifest in subtle behavioral alterations.

Among the first regions to be affected by neurofibrillary tangles is the entorhinal cortex (EC) (*10*), a hub for spatial navigation and memory. The EC contains spatially modulated cell types, including grid cells that fire at the vertices of equilateral triangles tiling the environment (*11*). In AD mouse models, early tauopathy in EC impairs grid cell functioning and spatial memory performance (*12*). In humans, grid cell activity can be indirectly measured as “grid-like representations” (GLRs) via functional magnetic resonance imaging (fMRI) (*13*–*15*). These GLRs were described to be functionally relevant for memory and spatial navigation (*4*, *13*, *16*). We previously showed evidence for impaired GLRs in young *APOE* ε4-carriers during virtual navigation in an arena (*4*). The spatial navigation performance of risk carriers was preserved, suggesting the use of compensatory strategies. Reduced GLRs were accompanied by relatively increased blood oxygenation level–dependent (BOLD) signals in the hippocampus (HC). Moreover, *APOE* ε4-carriers navigated more often at the border of the arena, possibly in an attempt to stabilize their GLRs (*4*, *5*, *17*).

Together, these findings suggest that subtle alterations in the neural and behavioral signatures of spatial navigation may occur in *APOE* ε4-carriers already at an early age, and these changes may constitute prime candidates for neurocognitive markers of AD (*1*). However, to dissect navigational strategies, potential compensatory mechanisms, and their underlying neural computations, it is crucial to probe the navigational function of grid cells more directly.

Theoretical considerations (*11*), computational models (*18*), and empirical studies in rodents (*19*) and humans (*16*) suggest that grid cells particularly support path integration (PI) processes. PI is the process of estimating one’s current position based on information about previous positions, heading direction, speed, and time elapsed. Specifically, grid cells may provide the computational basis for representing the integrated path (*18*) and for computing direct vectors to spatial goals (*20*, *21*). Accurate goal vectors are essential for the “incoming phase” of PI tasks in which subjects have to return to their home location.

When only information from visual flow is available, PI inevitably leads to error accumulation (*17*, *18*). This suggests that sparse environments and longer navigational routes unmask subtle PI deficits in *APOE* ε4-carriers. By contrast, when supportive spatial cues such as boundaries or landmarks are available [as in most previous studies, see (*4*, *5*)], additional brain regions could be recruited, in particular HC (*4*) and posterior cingulate/retrosplenial cortex (PC/RSC) (*21*, *22*). This may enable *APOE* ε4-carriers to perform on a par with control participants. In line with these predictions, our study unmasks a specific deficit of *APOE* ε4-carriers in PI when no supportive spatial cues are available and identifies the neural mechanisms underlying this deficit.

## RESULTS

### Experimental task and study sample

**Fig. 1:**
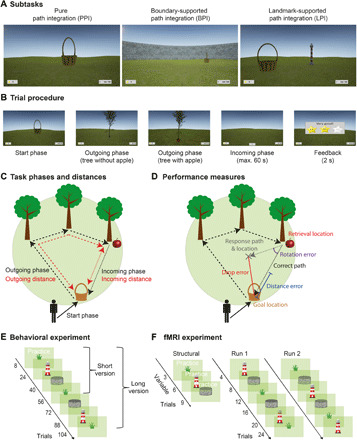
Experimental paradigm. (**A**) Participants performed a novel PI task (the Apple Game) in a virtual environment. The task comprised three subtasks that differed with regard to the presence or absence of supportive spatial cues: the PPI subtask without any supportive cue, the BPI subtask with a circular boundary, and the LPI subtask with an intramaze landmark (lighthouse) close to the center of the environment. (**B**) In each trial, participants collected a basket (start phase) and tried to remember its location (goal location). After navigating toward a variable number of trees (1 to 5; outgoing phase), which disappeared after having been reached, participants had to find their way back to the goal location (incoming phase). Last, they received feedback via different numbers of stars, depending on response accuracy. (**C**) Outgoing distance refers to the cumulated distance during the outgoing phase, and incoming distance refers to the Euclidean distance between retrieval location (tree with apple) and goal location (basket). (**D**) PI performance was assessed as the distance between the correct goal location and the response location (drop error). The drop error can be separated into the distance error (i.e., the difference between the retrieval-to-goal distance and the retrieval-to-response distance) and the rotation error (i.e., the difference between the retrieval-to-goal rotation and the retrieval-to-response rotation). (**E**) The behavioral task comprised 8 practice trials followed by 16 trials in each subtask (short version; in the long version, all subtasks were performed twice, resulting in 32 trials in each subtask). (**F**) The fMRI task consisted of up to nine practice trials during the structural scan, followed by two functional runs with six blocks of four trials each. See also fig. S1 and movie S1.

We assessed PI performance with a novel task, the “Apple Game” (Fig. 1 and fig. S1; Methods; see also movie S1). In each trial, participants first navigated to a basket (“start phase”) whose location they were instructed to remember (“goal location”). Next, participants navigated toward a variable number of trees (“outgoing phase”), systematically varying outgoing distance (and thereby PI difficulty), until they found a tree with an apple (“retrieval location”). Basket and trees appeared consecutively and disappeared as soon as the participant reached their locations. From the retrieval location, participants had to take the shortest route back to the basket location (incoming phase).

In different subtasks, participants either had to rely purely on visual flow [“pure PI” (PPI)] or were provided with supportive spatial cues in the form of a boundary [“boundary-supported PI” (BPI)] or an intramaze landmark [“landmark-supported PI” (LPI)]. PI performance was quantified via the distance between response location and correct location of the basket (“drop error”; for alternative metrics, see Fig. 1D).

We conducted the paradigm in *N* = 267 healthy participants genotyped for *APOE* across four different European sites (“*APOE* sample”; *n* = 65 *APOE* ε3/ε4-carriers, risk group; *n* = 202 *APOE* ε3/ε3-carriers, control group; age range, 18 to 75 years; mean age, 37.7 years; 38.6% male; table S1). Control and risk group did not differ regarding demographic characteristics or general cognitive status as indicated by mini-mental state examination [MMSE (*23*); table S2, fig. S1, and Supplementary Text]. All older participants (≥42 years, threshold established by means of kmeans clustering; see Methods) showed normal cognitive abilities (MMSE scores of ≥25). Additional analyses excluded an effect of *APOE* on time-counting strategies during the task, subjective navigation performance in everyday life, and pre-experimental navigation types (table S2 and Supplementary Text).

For a subgroup of this sample, structural MRI (sMRI) data were available, allowing us to establish relationships between behavioral performance and brain structure (“sMRI sample”; *n* = 99 participants; *n* = 23 risk carriers, *n* = 76 controls; table S3 and fig. S1). Furthermore, we recruited a separate group of *n* = 35 participants who completed a variant of the task inside the MR scanner (“fMRI sample”; table S1).

### Determinants of PI performance

We analyzed PI performance as a function of *APOE* genotype, subtask, and path distance in the *APOE* sample. We used a series of linear mixed models with “subtask” (PPI, BPI, or LPI) and “path distance” as within-subject variables and “*APOE*” as between-subject variable. Path distance refers to either “outgoing distance” (i.e., the accumulated path distance during the outgoing phase; Fig. 1C, model 1a, and Table 1; Methods) or “incoming distance” (i.e., the Euclidean distance between retrieval location and goal location; Fig. 1C, model 1b). When reporting inference statistics, “all” or “both” always refer to the two models with either one of the two path distances. “Subject” and “site” were added as random factors, and “sex” and “age” as covariates (for main effects and for interactions with subtask and genotype). Post hoc comparisons were Tukey-corrected for multiple comparisons (number of subtasks).

**Table 1:** Statistical models.

**Model**	**Criterion**	**Predictors (within** **subject)**	**Predictors (between** **subject)**	**Covariates**	**Random effects**
**Effects of *APOE*, subtask, and path distance on performance**					
1a	Performance	Subtask, outgoing distance	*APOE*	Age, sex	Subject, site
1b	Performance	Subtask, incoming distance	*APOE*	Age, sex	Subject, site
1c	Performance	Subtask, (outgoing and incoming) path distance, type of distance	*APOE*	Age, sex	Subject, site
**Effects of landmark and boundary**					
2a	Performance	Goal-to-boundary distance	*APOE*		Subject, site
2b	Performance	Goal-to-landmark distance	*APOE*		Subject, site
2c	Distance to boundary		*APOE*		Subject, site
2d	Distance to landmark		*APOE*		Subject, site
**Effects of EC volume, *APOE*, subtask, and path distance on performance**					
3a	Performance	Subtask, outgoing distance	*APOE*, relative EC volume	Age, sex	Subject
3b	Performance	Subtask, incoming distance	*APOE*, relative EC volume	Age, sex	Subject
**Effects of HC volume, *APOE*, subtask, and path distance on performance**					
4a	Performance	Subtask, outgoing distance	*APOE*, relative HC volume	Age, sex	Subject
4b	Performance	Subtask, incoming distance	*APOE*, relative HC volume	Age, sex	Subject
**Effects of PC/RSC volume, *APOE*, subtask, and distance on performance**					
5a	Performance	Subtask, outgoing distance	*APOE*, relative PC/RSC volume	Age, sex	Subject
5b	Performance	Subtask, incoming distance	*APOE*, relative PC/RSC volume	Age, sex	Subject
**Mechanistic model to explain path integration performance**					
6	Performance	Subtask, incoming distance	EC integrated path representations during incoming phase, HC goal proximity representations during incoming phase, pmEC GLRs, PC/RSC landmark representations		Subject

We found main effects of subtask and both path distance measures on PI performance (all *F* ≥ 466.42, all *P* < 0.001 for the two models with incoming and outgoing distance; fig. S2). Pairwise comparisons showed that performance was worse in the PPI as compared to the BPI and LPI subtasks (all *z* ≥ 22.25, all *P*_Tukey_ < 0.001). Performance in the BPI subtask was also worse than in the LPI subtask (both *z* ≥ 6.44, both *P*_Tukey_ < 0.001).

Notably, incoming distance had a more pronounced effect in the PPI subtask than in the two other subtasks, as shown by a significant subtask by incoming distance interaction (*F* = 99.17, *P* < 0.001; fig. S2). This was not the case for outgoing distance (*F* = 1.02, *P* = 0.361). Post hoc analyses confirmed that incoming distance had a stronger effect on performance in the PPI subtask than in the two other subtasks (both *z* ≥ 9.80, both *P*_Tukey_ < 0.001), with no difference between BPI and LPI (*z* = 1.11, *P*_Tukey_ = 0.511). Furthermore, we encountered significant main effects of sex and age (all *F* ≥ 63.49, all *P* < 0.001): Younger age and male sex predicted better performance [see fig. S2 (also for interactions involving covariates)]. These results show that the absence of supportive spatial cues not only reduces PI performance but also increases the impact of longer incoming distances, which is presumably related to larger error accumulation (*17*, *18*).

### Unmasking of *APOE* effects on PI in the absence of supportive spatial cues

**Fig. 2:**
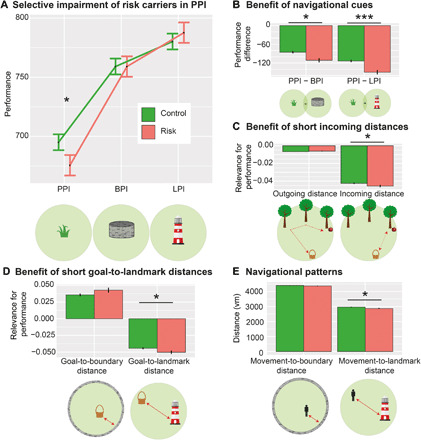
Performance as a function of genotype, distances, and subtask. (**A**) Performance (which is inversely related to drop error) is specifically impaired in risk carriers when no supportive spatial cues are available, i.e., in the PPI subtask. (**B**) Risk carriers benefit more from environmental landmarks and boundaries than controls. (A) and (B) depict results from model 1b; results from model 1a are statistically equivalent. (**C**) Incoming (but not outgoing) distance is more closely related to spatial memory performance in risk carriers than controls (model 1c). (**D**) Goal-to-landmark distance (but not goal-to-boundary distance) is more relevant in risk carriers than controls (models 2a and 2b). (**E**) Movement-to-landmark distance (but not movement-to-boundary distance) is significantly lower in risk carriers than controls (models 2c and 2d). *Y* axes show parameter estimates resulting from the different models; error bars, SEM; *P < 0.05, **P < 0.01, and ***P < 0.001. Control, *APOE* ε3/ε3-carriers; Risk, *APOE* ε3/ε4-carriers; PI, path integration; BPI, boundary-supported PI; LPI, landmark-supported PI; vm, virtual meters. See also figs. S1 to S3 and tables S1 and S2.

We next analyzed effects of *APOE* on performance. No main (subtask-independent) effect of *APOE* was observed (both *F* ≤ 0.04, both *P* ≥ 0.850), in line with a previous study showing generally unimpaired PI performance in *APOE* ε4-carriers (*5*). However, we observed a significant *APOE* by subtask interaction (both *F* ≥ 12.60, both *P* < 0.001; Fig. 2A), indicating different *APOE* effects on performance in the individual subtasks. The interaction was driven by worse performance of risk carriers as compared to controls specifically in the PPI subtask (both *z* ≥ 2.44, both *P*_Tukey_ ≤ 0.039), while there was no difference in the BPI (both *z* ≤ 0.05, both *P*_Tukey_ ≥ 0.998) or LPI subtasks (both *z* ≤ 0.98, both *P*_Tukey_ ≥ 0.593). To scrutinize this result, we compared performance as a function of *APOE* between the PPI subtask and either the BPI or LPI subtask (Fig. 2B). These contrasts measure a specific impairment of PI performance in the absence of supportive spatial cues. The performance of risk carriers declined more strongly than that of controls in the PPI subtask when compared to either the BPI subtask (both *z* ≥ 2.86, both *P*_Tukey_ ≤ 0.012) or the LPI subtask (both *z* ≥ 4.10, both *P*_Tukey_ < 0.001). There was no *APOE* effect on the performance difference between the BPI and LPI subtask (both *z* ≤ 1.26, both *P*_Tukey_ ≥ 0.410). Post hoc analyses revealed that the effect of genotype on performance was driven by increased rotation errors (Fig. 1D, fig. S3, and Supplementary Text).

Moreover, we observed an interaction between *APOE* and incoming distance (*F* = 3.94, *P* = 0.047; Fig. 2C), presumably reflecting stronger error accumulation (*17*, *18*) in risk carriers than controls. This effect was not significant for outgoing distance (*F* = 1.86, *P* = 0.173). *APOE* effects were significantly stronger for incoming than outgoing distance (three-way interaction between outgoing distance, incoming distance, and *APOE* in a model including both distance types; *F* = 4.68, *P* = 0.030; model 1c). This indicates that performance in risk carriers is particularly affected by the incoming (i.e., Euclidean) distance between retrieval and goal location.

Because risk carriers’ performance was on par with controls when supportive spatial cues were present, we hypothesized that the distance to these cues determines their performance more than in controls. We thus investigated PI performance as a function of “spatial cue distance” (i.e., the distance between the goal location and the boundary or the landmark; models 2a and 2b). Generally, higher distances from the boundary and lower distances from the landmark were associated with higher performance (main effects of goal-to-boundary distance and goal-to-landmark distance: both *F* > 342.45, both *P* < 0.001). Better PI performance in trials with higher distances between the goal location and the environmental boundary may be counterintuitive, because boundaries constitute an additional visual cue. However, goal locations in the center of the environment have a lower range of possible drop errors than goal locations in the periphery of the environment, leading to lower drop errors per se, as described previously (*24*).

In addition, the performance of risk carriers declined more strongly with increasing goal-to-landmark distance than the performance of controls (interaction effect between goal-to-landmark distance and *APOE*: *F* = 4.55, *P* = 0.033; model 2b; Fig. 2D). By contrast, goal-to-boundary distance did not exhibit different effects in risk and control participants (interaction effect between goal-to-boundary distance and *APOE*: *F* = 2.59, *P* = 0.107; model 2a; Fig. 2D).

Irrespective of PI performance, we investigated the mean distance of participants’ navigation to the boundary or the landmark (only during the incoming phase, as movements during the outgoing phase were determined by the trees; models 2c and 2d). This revealed that risk carriers showed a different navigational pattern: They navigated in closer proximity to the landmark (*F* = 6.20, *P* = 0.013; model 2d; Fig. 2E), suggesting that they used the landmark information to a higher degree to guide their behavior. By contrast, risk carriers navigated at similar distances to the boundary as controls (*F* = 1.35, *P* = 0.245; model 2c, Fig. 2E).

In summary, a selective PI deficit in *APOE* ε4-carriers was unmasked when environments lacked spatial cues and participants had to rely purely on PI. When environmental landmarks were available, risk carriers relied more strongly on their proximity.

### Brain structural determinants of *APOE* effects on PI

How is performance in the different subtasks related to brain volume in areas relevant for spatial navigation, and how are these relationships modulated by *APOE*? We analyzed PI performance as a function of relative gray matter volume in three regions of interest (ROIs): EC, HC, and PC/RSC. We used mixed models with subtask and path distance (outgoing distance, models 3a to 5a; or incoming distance, models 3b to 5b) as within-subject variables, *APOE* and “volume” of one of the three ROIs as between-subject variables, and subject as random factor (sMRI sample; Table 1 and Methods). Age and sex were added as covariates. Because these models contain two continuous predictors of interest (path distance and volume), post hoc comparisons were performed on quintiles of the path distance predictor (Tukey-corrected for number of compared quintiles). Qualitatively identical results were obtained when the path distance predictor was subdivided into quartiles or tertiles (Supplementary Text).

**Fig. 3:**
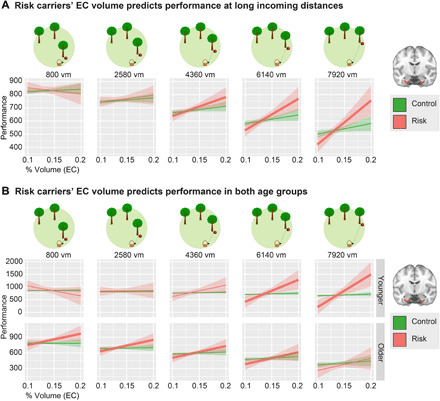
Performance as a function of genotype, age, incoming distance, and EC gray matter volume. (**A**) EC gray matter volume predicts performance only during PI with long incoming distances (middle to right panels) and only in risk carriers (model 3b). (**B**) In younger risk carriers, EC gray matter volume predicted performance during PI trials with long incoming distances. In older risk carriers, EC gray matter volume predicted performance during the majority of all trials (model 3b). The young group comprises subjects aged 18 to 28 years; the older group contains subjects aged 53 to 75 years (see age histogram in fig. S1C). As the models contained two continuous predictors, one of them (incoming distance) was discretized into quintiles for post hoc tests and graphical depiction. Thicker lines mark slopes that are significantly different from zero. *Y* axes show parameter estimates resulting from the different models; shaded areas, SEM. Control, *APOE* ε3/ε3-carriers; Risk, *APOE* ε3/ε4-carriers; % volume, percent of whole-brain volume. See also fig. S1 and tables S1 and S3.

Risk carriers and controls did not differ regarding age, sex, whole-brain volume, and relative volume in any ROI (table S3 and fig. S1), in line with previous findings (*25*). Also, relative ROI volumes did not directly predict performance in any subtask, for either controls or risk carriers (all *z* ≤ 1.68, all *P*_Bonferroni_ ≥ 0.279). However, an effect of EC volume on performance in risk carriers was unmasked when analyzed in relationship to incoming distance: When we examined the effect of EC volume on performance (model 3b), we encountered a significant interaction with *APOE* and incoming distance (*F* = 6.70, *P*_Bonferroni_ = 0.030; Fig. 3A). While EC volumes did not predict performance for shorter incoming distances in either risk carriers or controls [800 to 2580 virtual meters (vm): all *z* ≤ 1.24, all *P*_Tukey_ ≥ 0.214], they exerted a significant effect for longer incoming distances specifically in risk carriers (4360 to 7920 vm: all *z* ≥ 2.17, all *P*_Tukey_ ≤ 0.030), but not in controls (all *z* ≤ 1.87, all *P*_Tukey_ ≥ 0.061). Effects in risk carriers were significantly larger than in controls at large incoming distances (7920 vm: *z* = 2.56, *P*_Tukey_ = 0.044), but not at lower incoming distances (800 to 6140 vm: all *z* ≤ 2.13, all *P*_Tukey_ ≥ 0.126). No comparable effects were found for outgoing distance (model 3a), in HC (model 4a/4b), or PC/RSC (model 5a/5b). These results show a selective influence of EC volume on performance in risk carriers for longer incoming distances, suggesting that effects of subtle structural degradation of the EC in risk carriers become visible in situations of large potential error accumulation.

### Age-related modulations of *APOE* effects on PI performance

Age was included as a covariate in models 1a to 1c, and we described interactions with age in fig. S2. Notably, we found a significant subtask by *APOE* by age interaction (*F* = 5.79, *P* = 0.003; fig. S2F), suggesting that the difference between risk carriers and controls in the PPI condition might be modulated by age. However, risk carriers did not differ from controls with respect to age-related decline of performance in any of the subtasks (all *z* < 1.79, all *P*_Tukey_ = 0.171).

We nevertheless aimed at a more detailed understanding of the age-related modulations of our *APOE* effects, which is crucial to evaluate the possible implications of our findings for preclinical dementia diagnosis. We therefore split the *APOE* sample into a “younger” subsample (*n* = 163; mean age ± SD, 24.32 ± 4.87) and an “older” subsample (*n* = 104; mean age ± SD, 58.71 ± 7.75) in a data-driven manner using MATLAB’s kmeans clustering algorithm. This method resulted in a cutoff age of 42 years.

The proportion of risk carriers did not differ between the younger (risk group, *n* = 35; control group, *n* = 128) and older age group [*n* = 30; *n* = 74; χ^2^(1) = 1.87, *P* = 0.190]. The proportion of male and female participants also did not differ between the two age groups (χ^2^ = 0.55, *P* = 0.269).

We then re-performed all analyses separately for the younger and older group. First, we examined the results of model 1 in detail. In both age groups, we found main effects of subtask and both path distance measures on PI performance (all *F* ≥ 77.76, all *P* < 0.001). Pairwise comparisons showed that performance in the PPI subtask was worse than in the BPI and LPI subtasks (all *z* ≥ 10.69, all *P*_Tukey_ < 0.001). Performance was better in LPI than in BPI (both *z* ≥ 2.35, both *P*_Tukey_ ≤ 0.049). In both groups, we encountered a significant subtask by incoming distance interaction (both *F* ≥ 27.68, both *P* < 0.001), but no subtask by outgoing distance interaction (both *F* ≤ 1.10, both *P* ≥ 0.334). Incoming distance had a stronger effect on performance in the PPI subtask than in the two other subtasks (all *z* ≥ 6.13, all *P*_Tukey_ < 0.001), and there was no difference between BPI and LPI (both *z* ≤ 1.85, both *P*_Tukey_ ≥ 0.154). Moreover, we found a significant main effect of sex in both age groups (with females performing worse; all *F* ≥ 6.56, all *P* ≤ 0.012). Furthermore, we also found significant main effects of age in both subsamples (all *F* ≥ 4.99, all *P* ≤ 0.028). These effects replicate the findings in the entire sample and thus demonstrate that the age distribution of our sample did not significantly influence the main results of our study.

**Fig. 4:**
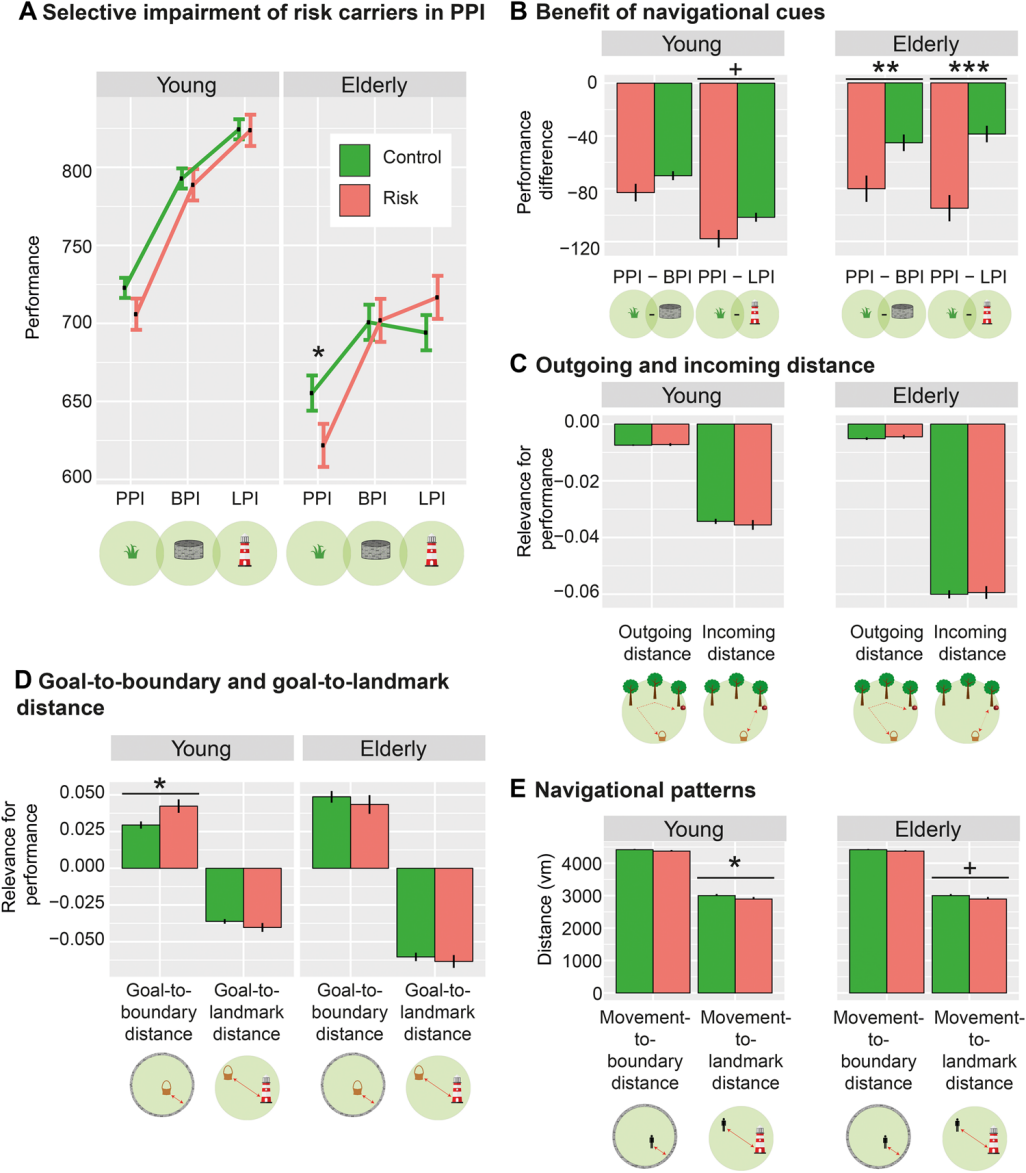
Performance as a function of genotype, distances, and subtask split by age groups. The younger age group comprises subjects aged 18 to 41 (*n* = 163), and the older age group comprises subjects aged 42 to 75 (*n* = 104). (**A**) Performance (which is inversely related to drop error) is specifically impaired in older risk carriers when no supportive spatial cues are available, i.e., in the PPI subtask. (**B**) In older participants, risk carriers benefit more from environmental landmarks and boundaries than controls. (A) and (B) depict results from model 1b; results from model 1a are statistically equivalent. (**C**) In both age groups, neither incoming nor outgoing distance is more closely related to spatial memory performance in risk carriers than controls. (**D**) In the younger age group, goal-to-boundary distance is more relevant in risk carriers than in controls (models 2a and 2b). (**E**) In younger participants, movement-to-landmark distance (but not movement-to-boundary distance) is significantly lower in risk carriers than in controls (models 2c and 2d). *Y* axes show parameter estimates resulting from the different models; error bars, SEM. ^+^*P* < 0.10, *P < 0.05, **P < 0.01, and ***P < 0.001. Control, *APOE* ε3/ε3-carriers; Risk, *APOE* ε3/ε4-carriers. See also figs. S1 to S3 and tables S1, S2, and S4.

In both age groups, we found no significant main effect of *APOE* (all *F* ≤ 0.10, all *P* ≥ 0.756) but observed significant *APOE* by subtask interactions on PI performance (all *F* ≥ 3.95, all *P* ≤ 0.019; Fig. 4A). These effects again replicate the findings in the entire sample. Pairwise comparisons revealed that the performance of risk carriers in the PPI subtask was significantly worse than the performance of controls only in the older age group (both *z* ≥ 2.72, both *P*_Tukey_ ≤ 0.018), but not in the younger age group (both *z* ≤ 1.70, both *P*_Tukey_ ≥ 0.206). This result suggests that the adverse effect of *APOE* ε4 on PI performance exacerbates with increasing age.

In both age groups, we did not observe a difference between risk carriers and controls when comparing PI performance in the BPI versus the LPI subtask (all *z* ≤ 1.86, all *P*_Tukey_ ≥ 0.151). The younger age group showed no difference between risk carriers and controls when comparing PI performance in the PPI versus the BPI subtask (both *z* ≤ 1.72, both *P*_Tukey_ ≥ 0.197; Fig. 4B). There was a trend for a difference between risk carriers and controls when comparing PI performance in the PPI versus the LPI subtask (both *z* ≥ 2.06, both *P*_Tukey_ ≤ 0.099). In the older age group, risk carriers performed significantly worse in the PPI subtask as compared to both the BPI subtask and the LPI subtask (all *z* ≥ 2.96, all *P*_Tukey_ ≤ 0.009: Fig. 4B). These results show again that the adverse effect of *APOE* ε4 on PI performance exacerbates with increasing age.

We did not observe any *APOE* by incoming distance interactions in either age group (both *F* ≤ 0.61, both *P* ≥ 0.436; Fig. 4C), presumably due to reduced statistical power. Model 1c, in which we analyzed three-way interactions between outgoing distance, incoming distance, and *APOE*, was thus not applied.

Next, we examined the results from model 2 in detail, which assessed the effects of boundary and landmark distance from the goal location on PI performance. Higher distances from the boundary and lower distances from the landmark were associated with better performance in both age groups (all *F* ≥ 198.03, all *P* < 0.001), replicating our previous results. We did not observe the significant *APOE* by goal-to-landmark distance effect in either age group (both *F* ≤ 1.47, both *P* ≥ 0.225; Fig. 4D), presumably due to reduced statistical power in the subsamples. Instead, we observed a significant interaction of *APOE* with goal-to-boundary distance in the young sample only (*F* = 6.25, *P* = 0.012; old sample: *F* = 0.49, *P* = 0.485; Fig. 4D), suggesting that young risk carriers benefit more from central positions of goal locations.

Last, young risk carriers navigated closer to the landmark (*F* = 3.96, *P* = 0.047) and older risk carriers showed a trend for this effect (*F* = 3.02, *P* = 0.085; Fig. 4E), largely replicating the result from the entire sample. We did not encounter differences in the movement-to-boundary distance between genotypes (both *F* ≤ 1.20, both *P* ≥ 0.274; Fig. 4E). We present a synopsis of all results in table S4.

In addition, we tested whether there was an inverse U-shaped effect of *APOE* across the life span, by adding a quadratic age variable to models 1a and 1b (“age^2^”). Adding age^2^ did not improve model fit [both χ^2^(1) ≤ 0.22, both *P* ≥ 0.635]. We found no main effect of age^2^ (both *F* ≤ 0.81, both *P* ≥ 0.370) or interactions with subtask or *APOE* (all *F* ≤ 1.76, all *P* ≥ 0.171), suggesting that there were no inverse U-shaped effects of *APOE* across the life span.

In summary, these analyses show that performance differences between subtasks, effects of incoming distance, and the interaction between subtask and incoming distance are present in both age groups. We found significant subtask by *APOE* interactions in both age groups, with a pattern of results replicating the findings in the entire cohort. We found that the adverse effect of *APOE* ε4 on PPI performance was present in the older subsample only, suggesting that this *APOE* effect may be mediated by age-dependent accumulation of preclinical AD pathology. This interpretation prompts future studies that assess whether PI deficits are related to AD biomarkers.

Some effects did not replicate in the individual age groups: There was no interaction between *APOE* and incoming distance (i.e., a stronger reduction in performance with larger incoming distances for risk carriers as compared to controls), and there was no interaction between *APOE* and goal-to-landmark distance (i.e., a stronger reduction in performance with larger distances between goal and landmark in risk carriers as compared to controls). This is probably due to lower statistical power in each subsample. The younger subsample showed a significant *APOE* by goal-to-boundary distance interaction that was not present in the entire cohort (i.e., risk carriers showed a more pronounced performance increase in trials with more central goal locations).

Next, we re-performed the sMRI analysis after splitting the sMRI sample into two age groups. This was done according to the split that is apparent from the age histogram (young group: ≤28 years, *n* = 48; mean age ± SD, 22.42 ± 2.28; older group: ≥53 years, *n* = 51; mean age ± SD, 63.22 ± 5.42; fig. S1C). The proportion of risk carriers did not differ between the younger (risk group, *n* = 9; control group, *n* = 39) and older (*n* = 14; *n* = 37) age group [χ^2^(1) = 1.05, *P* = 0.348]. The proportion of male and female participants also did not differ between the two age groups [χ^2^(1) = 0.01, *P* ≈ 1.000].

Similar to our main analysis, EC volume did not predict performance in either the older group or the younger group (all *F* ≤ 2.07, all *P* ≥ 0.157). In the older group, we observed a trend for a three-way interaction between incoming distance, *APOE*, and relative EC volume (*F* = 3.60, *P* = 0.058; Fig. 3B), similar to what we had found in the entire sample. This effect was not present in the younger group (*F* = 0.29, *P* = 0.593).

To understand the data in detail, we conducted post hoc tests on quintiles of incoming distances. In control participants, PI performance did not depend on EC volume in either age group (all *t* ≤ 1.22, all *P*_Tukey_ ≥ 0.221). By contrast, and as in the entire sample, risk carriers exhibited significant relationships between EC volume and performance at various incoming distances (see Fig. 3B). In younger risk carriers, EC volume was positively related to performance at high incoming distances (>4360 vm: both *t* ≥ 3.10, both *P*_Tukey_ ≤ 0.002). In older risk carriers, positive relationships between EC volume and performance occurred at almost all incoming distances (<7920 vm: all *t* ≥ 2.10, all *P*_Tukey_ ≤ 0.036). Because we did not observe a three-way interaction between EC volume, genotype, and incoming distance for either age group (see above), these differences should be considered cautiously, however.

Thus, these analyses confirm that EC volume predicted PI performance specifically in risk carriers, underscoring that their EC integrity is of particular importance for PI performance. In conclusion, while most *APOE* effects occurred across the entire age range, the adverse effect of *APOE* ε4 on PPI seemed to be confined to older participants, underlining the relevance of our findings for detecting older persons at risk for cognitive vulnerability and AD.

### fMRI correlates of integrated path and goal proximity during PI

To understand the neural basis of PI and the impact of supportive spatial cues in our paradigm, we conducted an fMRI study with an adapted version of the task (fMRI sample; see Methods). Because we were interested in the physiological fMRI correlates of our PI task, the fMRI sample comprised a group of young and healthy participants, which were recruited irrespective of *APOE* genotype. First, we analyzed neural representations of two fundamental metrics of PI: the instantaneous integrated path (i.e., the length of the travelled path during either the outgoing or incoming phase) and the instantaneous goal proximity (i.e., the distance between the subject’s current location and the goal). We created a general linear model (GLM) with regressors for the entire time of movement during the outgoing and incoming phase, separately for each subtask (fig. S4). In two separate analyses, we included either integrated path or goal proximity as time-varying parametric modulators during movement (sampled at a temporal resolution of 5 Hz). We tested how these measures of PI were related to BOLD activity in the three ROIs (EC, HC, and PC/RSC). PC/RSC was defined as the posterior-ventral part of the cingulate gyrus [following (*26*, *27*)], resulting in a mask slightly larger than RSC proper. *P* values were false discovery rate (FDR)–corrected for multiple comparisons (number of ROIs and number of contrasts).

**Fig. 5:**
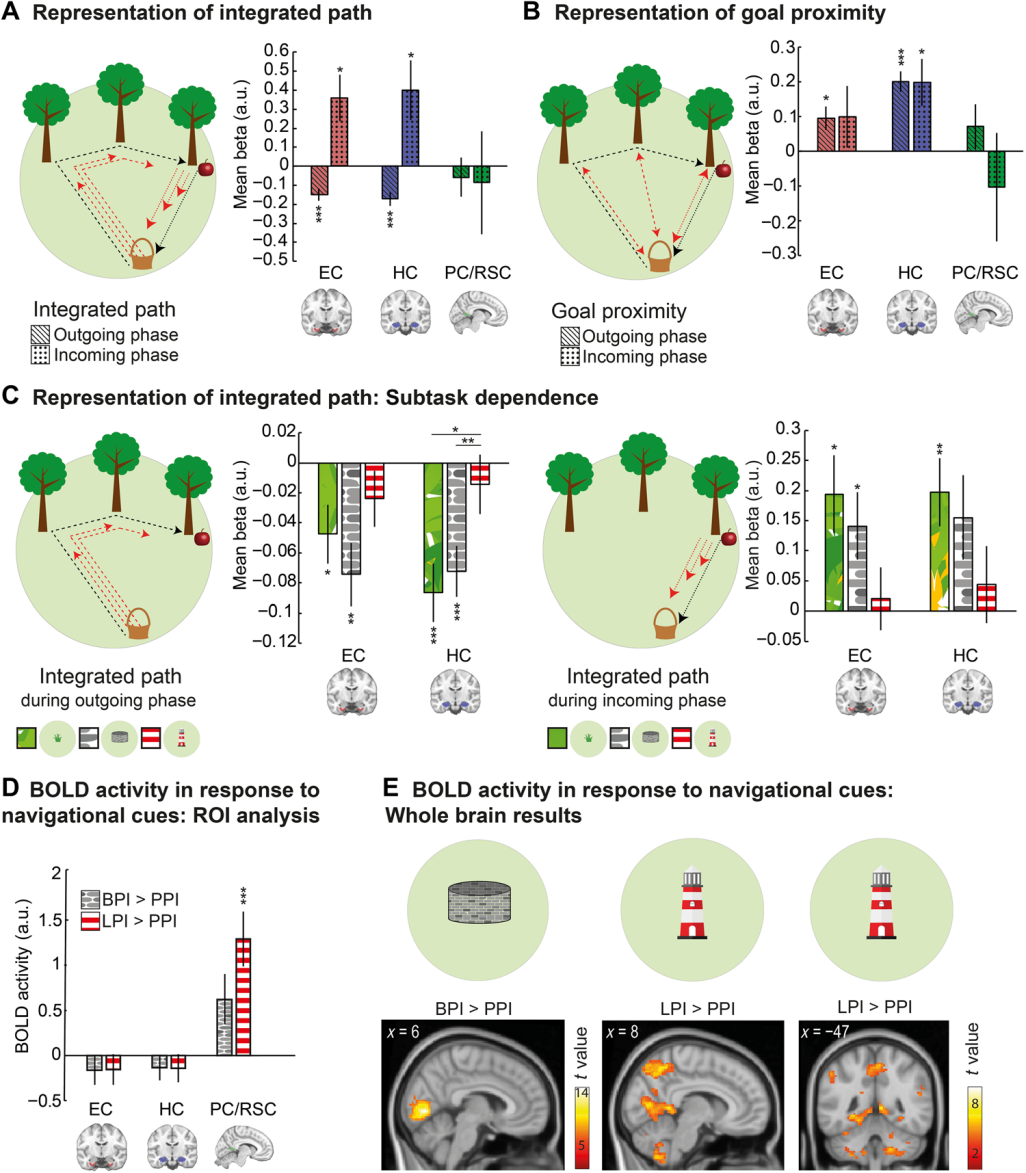
fMRI results: Neural determinants of integrated path, goal proximity, and subtask. (**A**) Representation of integrated path in EC and HC: Deactivation during navigation at longer integrated paths during outgoing phase and activation during incoming phase. (**B**) Representation of goal proximity: Activation for navigation closer to the goal in HC in both trial phases and in EC during the outgoing phase. (**C**) Subtask dependence of integrated path representations: HC deactivation during navigation at longer integrated paths during the outgoing phase was modulated by subtask. During the incoming phase, we observed a statistical trend for a similar result in EC and HC. (**D**) Significantly higher activity in PC/RSC during the LPI as compared to the PPI subtask. (**E**) Confirmatory whole-brain analyses: (left) activation of visual areas during the BPI as compared to the PPI subtask and (right) higher PC/RSC activity in the LPI than in the PPI subtask. *P* values are FDR-corrected for the number of contrasts and for the number of ROIs in all ROI analyses (A, B, and D). For post hoc comparisons between subtasks in (C), *P* values are FDR-corrected for number of subtasks. *P < 0.05, **P < 0.01, and ***P < 0.001. Error bars (A to D), SEM. Statistical parametric maps in (E) are thresholded at *P* < 0.05, family-wise error (FWE)–corrected for whole brain (left) and small volume–corrected for PC/RSC (middle and right), and clusters are considered significant at *P* < 0.05, FWE-corrected. FDR, false discovery rate; a.u., arbitrary units. See also figs. S4 and S5 and table S5.

During the outgoing phase, EC and HC showed pronounced deactivation with increasing integrated path (both *t*_34_ ≥ 4.68, both *P*_FDR_ < 0.001; Fig. 5A). This was reversed during the incoming phase, where we observed increased activation in both areas with integrated path (both *t*_34_ ≥ 2.55, both *P*_FDR_ ≤ 0.046; Fig. 5A). When analyzed separately for the different subtasks, we found that EC and HC activity levels always differed significantly from zero in the PPI subtask (Fig. 5C and Supplementary Text). By contrast, integrated path did not predict activity in the LPI subtask, suggesting that this representation is less relevant in this subtask. Activity in PC/RSC did not show any relationship with integrated path.

During both the outgoing and incoming phases, HC activity increased with goal proximity (both *t*_34_ ≥ 2.96, both *P*_FDR_ ≤ 0.028; Fig. 5B). In EC, we observed this effect only during the outgoing phase (*t*_34_ = 2.86, *P*_FDR_ = 0.029), but not during the incoming phase (*t*_34_ = 1.12, *P*_FDR_ = 0.813). No relationship between goal proximity and PC/RSC activity was observed.

As integrated path and goal proximity are typically related in the real world and strongly correlated in our task as well (ρ = −0.38, *Z* = 3.72, *P* < 0.001), we included them in two separate GLMs. We conducted a control analysis to demonstrate that representations of integrated path were not simply side effects of goal proximity representations by including both parametric modulators into a single GLM (see Methods). Results were highly similar (Supplementary Text).

Together, these results show that BOLD responses in EC and HC represent integrated path and goal proximity, two related variables that are crucial for solving PI tasks. This is in accordance with fMRI studies in humans [e.g., (*20*)], showing representations of goal direction and distance in EC and HC.

### fMRI correlates of spatial information from boundaries and landmarks

Next, we analyzed which brain regions are recruited by supportive spatial cues. We used a GLM with “task phase” (start phase, outgoing phase, incoming phase, feedback) and subtask (PPI, BPI, and LPI) as regressors (fig. S5) and contrasted activity during the PPI subtask with activity during the BPI or LPI subtasks. In PC/RSC, activity increased during the LPI as compared to the PPI subtask (*t*_34_ = 4.27, *P*_FDR_ < 0.001; Fig. 5D) and did not differ between the BPI versus the PPI subtask (*t*_34_ = 2.24, *P*_FDR_ = 0.095). Activity in EC and HC did not differ between the subtasks (all *t*_34_ ≤ 0.97, all *P*_FDR_ ≥ 0.385).

An exploratory whole-brain analysis confirmed these results and additionally showed higher activity in V1 and adjacent visual areas during the BPI and LPI subtasks, probably due to the higher amount of visual information (Fig. 5E; for all clusters, see table S5). Hence, PC/RSC appears to be specifically involved in processing spatial information derived from environmental landmarks, in line with previous results (*21*, *22*).

### Support of PI computations by fMRI grid-like representations

**Fig. 6:**
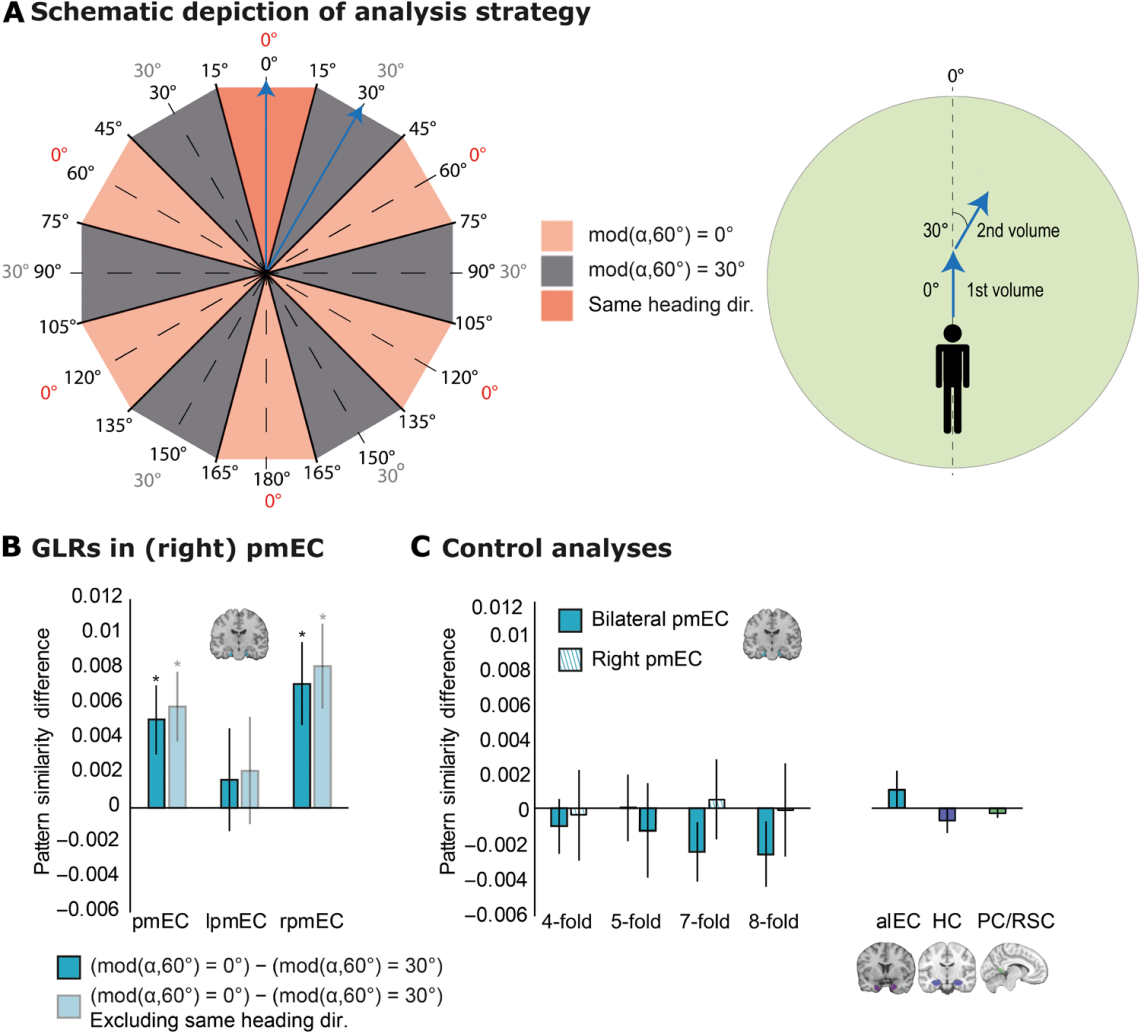
Grid-like representations in pmEC. (**A**) Left: Schematic depiction of angular differences in 360° space (inner numbers) and in 60° space (outer numbers). We expected higher pattern similarity for angular differences of mod(α,60°) = 0° (rose, where α is the angular difference between two movement directions) as compared to angular differences of mod(α,60°) = 30° (gray). We expected the same result when excluding pattern similarities of the same heading direction (dark rose). Right: Movement directions of two exemplary fMRI volumes (blue arrows). In the first volume, the subject navigates at an angle of 0° with respect to the reference axis. In the second volume, the subject navigates at an angle of 30°. This results in an angular difference of 30° (in 360° and 60° spaces) and thus mod(α,60°) = 30° (compare to blue arrows on the left). (**B**) In bilateral and in right pmEC, pattern similarity for angular differences of mod(α,60°) = 0° was significantly higher than pattern similarity for angular differences of mod(α,60°) = 30°, suggesting a hexadirectional symmetry of pattern similarities (dark blue bars). Same result when removing movements with similar heading directions in 360° space (light blue bars). (**C**) Control analyses. Left: No evidence for fourfold, fivefold, sevenfold, or eightfold rotational symmetry of pattern similarity in bilateral or right pmEC. Right: No evidence for sixfold rotational symmetry of pattern similarity in alEC, HC, and PC/RSC. Error bars (B and C), SEM. *P < 0.05. mod, modulus; dir., direction; pmEC, posterior-medial entorhinal cortex; alEC, anterior-lateral entorhinal cortex; HC, hippocampus.

Previous fMRI studies established a hexadirectional modulation of BOLD activity in the EC as a macroscopic signature of grid cell activity (*4*, *13*, *14*). We thus analyzed GLRs in our paradigm using a multi-voxel pattern approach (see Methods) (*14*). We focused on the posterior-medial EC (pmEC), the putative human homolog of the rodent medial EC (*11*, *14*, *28*). The analysis is based on the rationale that the hexadirectionally symmetric firing pattern of grid cells results in higher pattern similarity when movement directions are 60°, 120°, 180°, etc. apart from each other than when they have an offset of 30°, 90°, 150°, etc. (Fig. 6A).

We observed a significant hexadirectional symmetry of pattern similarity in pmEC (*t*_34_ = 2.56, *P* = 0.015; Fig. 6B). A post hoc test revealed that the effect was confined to right pmEC (*t*_34_ = 2.98, *P* = 0.005), in accordance with previous studies on GLRs (*4*, *13*). GLRs were not driven by a head direction signal: Eliminating movement directions with angular differences of −15° to 15° still resulted in a hexadirectional signal (bilateral pmEC: *t*_34_ = 2.90, *P* = 0.006; right pmEC: *t*_34_ = 3.36, *P* = 0.002; Fig. 6B). We performed a series of control analyses to ensure validity and specificity of the GLRs (Fig. 6C and Supplementary Text).

### Predictions of PI performance by neural representations of spatial features

**Fig. 7:**
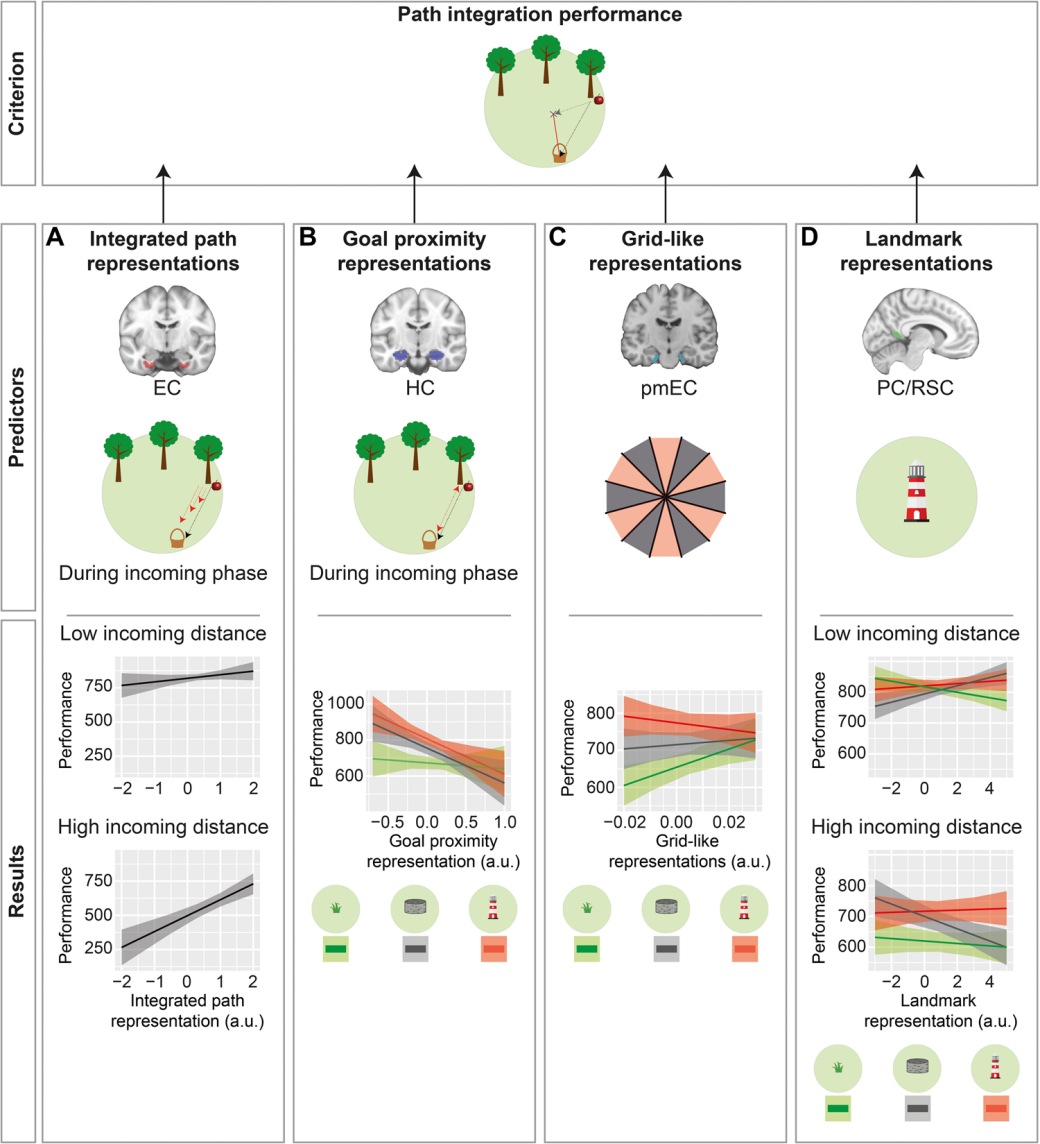
Mechanistic model to predict PI performance. We aimed at predicting PI performance as a function of fMRI-based representations of spatial features in combination with subtask and incoming distance (model 6). (**A**) Integrated path representations in EC interacted with incoming distance in predicting PI performance: At higher incoming distances, stronger integrated path representations in EC were associated with better performance. (**B**) Goal proximity representations in HC interacted with subtask in predicting PI performance. In none of the subtasks was the prediction significant by itself. (**C**) GLRs in pmEC interacted with subtask in predicting PI performance: Only in PPI, higher GLRs in pmEC were associated with better performance. (**D**) Landmark representations in PC/RSC interacted with subtask and incoming distance in predicting PI performance. In none of the individual subtask by incoming distance combinations was the prediction significant by itself. As the model contained two continuous predictors, one of them (incoming distance) was discretized into quintiles for post hoc tests and for graphical depiction; only quintiles 1 and 5 are depicted (A and D). *Y* axes show parameter estimates for performance; shaded areas, SEM.

Last, we aimed at establishing a mechanistic model that could explain PI performance (Fig. 7). We built an exploratory mixed linear model with subtask and incoming distance as within-subject predictors, subject as random factor, and the following fMRI-based between-subject predictors: (i) EC representations of integrated path during the incoming phase, (ii) HC representations of goal proximity during the incoming phase, (iii) GLRs in pmEC, and (iv) PC/RSC landmark representations (model 6, Table 1). The other potential fMRI-based predictors were not included into the model because they either caused multicollinearity or did not significantly improve model fit (for a detailed explanation of the model buildup, see Methods). We allowed interactions of the fMRI-based predictors with subtask or incoming distance, but not with the other fMRI-based predictors. Post hoc comparisons were Tukey-corrected for multiple comparisons (number of subtasks or number of compared quintiles).

First, we found that EC representations of integrated path during the incoming phase predicted performance (*F* = 16.19, *P* < 0.001). This effect was more pronounced at high incoming distances, as shown by a significant interaction with incoming distance (*F* = 5.43, *P* = 0.020): At higher incoming distances (quintiles 2 to 5), EC representations of integrated path showed a significant association with performance (all *z* ≥ 3.32, all *P*_Tukey_ < 0.005). This was not the case at low incoming distances (quintile 1: *z* = 1.97, *P*_Tukey_ = 0.221).

Second, while we did not encounter a significant main effect of HC representations of goal proximity during the incoming phase (*F* = 1.39, *P* = 0.247), we found a significant interaction with subtask (*F* = 3.43, *P* = 0.032). However, post hoc tests revealed no significant association between this fMRI predictor and performance in any subtask (all *z* ≤ 2.08, all *P*_Tukey_ ≥ 0.109).

Third, the magnitude of GLRs had no main effect on performance (*F* = 0.73, *P* = 0.398) but showed a significant interaction with subtask (*F* = 7.98, *P* < 0.001). Post hoc comparisons revealed that GLRs predicted performance to a greater extent in PPI than in LPI (*z* = 3.99, *P*_Tukey_ < 0.001). No differences were encountered between the other subtask combinations (both *z* ≤ 2.14, both *P*_Tukey_ ≥ 0.082). When analyzed separately, we found that GLRs significantly predicted performance in the PPI subtask (*z* = 2.52, *P*_Tukey_ = 0.035), but not in the other subtasks (both *z* ≤ 0.96, both *P*_Tukey_ ≥ 0.711). These results indicate that the grid cell system in pmEC supports PI specifically in the absence of supportive spatial cues.

Last, the magnitude of PC/RSC representations of landmarks showed no main effect on performance (*F* = 0.07, *P* = 0.793), but we encountered a significant two-way interaction with subtask (*F* = 6.93, *P* = 0.001) and a three-way interaction with subtask and incoming distance (*F* = 4.36, *P* = 0.013). However, PC/RSC representations of landmarks predicted performance in none of the individual follow-up tests (all *z* ≤ 1.47, all *P*_Tukey_ ≥ 0.543).

## DISCUSSION

Our behavioral results show that *APOE* ε4-carriers perform as well as controls as long as they can use supportive spatial information from a boundary or a landmark. Their deficit in PI is unmasked, however, when potentials for error accumulation increase (i.e., with higher PI distance) and/or when no compensatory strategies can be used. In these conditions, PI performance relates to EC gray matter volume and to the strength of entorhinal GLRs. Supportive spatial cues improve performance, presumably via compensatory recruitment of other regions including the PC/RSC.

We propose that changes in grid cell functioning underlie the impaired performance of risk carriers during PPI because various studies identified grid cells as the main neural substrate for PI (*16*, *19*). In our fMRI study, we observed a positive relationship between the strength of GLRs and PI performance exclusively in the absence of supportive spatial cues that could reduce error accumulation during PI computations (*17*, *18*). This indicates that the experimental condition in which risk carriers showed impaired PI performance relied most strongly on the grid cell system. Risk carriers may thus experience higher error accumulation during PPI due to a compromised EC grid cell system.

Furthermore, we found representations of integrated path and goal proximity in EC and HC during the outgoing as well as during the incoming phase. This result is in line with models suggesting that EC grid cells provide the computational basis for representing the integrated path (i.e., the distance and direction traveled) (*18*). A complementary model suggests that grid cells enable the computation of a direct vector to the goal (*20*, *21*). Accordingly, we demonstrated that risk carriers’ performance is particularly affected by high incoming distances and that this is associated with a higher dependency on EC volume in *APOE* ε4-carriers.

An environmental boundary or a landmark allowed risk carriers to perform as well as controls. This is consistent with a recent study showing similar PI performance in risk and control participants in rich virtual environments (*5*). This previous study examined the effect of *APOE* genotype on wayfinding distance and wayfinding duration during map-based navigation and on rotation errors during PI (“flare accuracy”). A particular strength of this study was the use of normative benchmark big data from the Sea Hero Quest (SHQ) game that was conducted by more than 27,000 participants worldwide (*5*, *29*). The authors demonstrated that *APOE* ε4-carriers exhibited larger wayfinding distances than noncarriers, whereas wayfinding duration and flare accuracy were similar (*5*). Given that wayfinding distance may particularly rely on grid cell–based navigational processes, the study is in line with the notion that grid cell dysfunction may constitute a sensitive biomarker for AD risk, consistent with the current study.

The new results presented here are complementary to this earlier study in several respects: First, effects of *APOE* ε4 were observed during map-based wayfinding in the study by Coughlan and colleagues, whereas the current study found a detrimental effect of *APOE* ε4 on PPI. Thus, different navigational processes were analyzed. Second, the current study applied an adapted version of the behavioral paradigm during fMRI scanning, which allowed us to measure the role of GLRs in our task and also to assess the recruitment of other navigational systems. In addition, structural neuroimaging allowed us to relate the behavioral results to EC volumes. We could thus identify a putative neural correlate for the PI deficit found in our study, i.e., GLRs in EC. In the future, it will be pivotal to directly compare the neurocognitive processes during the different experimental tasks and to further disentangle the effects of *APOE* genotype and AD pathology on different subcomponents of navigational behavior.

Using fMRI, we demonstrated increased PC/RSC activity in response to an environmental landmark, consistent with previous studies (*22*). The PC/RSC provides a neural basis for viewpoint-dependent representations of local place and direction (*21*, *22*), which may be at the core of compensatory strategies used by risk carriers. We propose that risk carriers recruited the PC/RSC in the landmark condition to a greater extent than controls to counteract error accumulation during PI and to compensate for a reduced reliability of GLRs, thus anchoring the cognitive map (*21*, *30*). In support of this idea, risk carriers showed a stronger relationship between goal-to-landmark distance and performance, and navigated in closer proximity to the landmark during the incoming phase. However, future fMRI studies with genotyped participants are required to corroborate this idea.

In the BPI subtask, the relationship between performance and goal-to-boundary distance was not modulated by genotype, and risk carriers did not navigate closer to the boundary as compared to control participants. This null finding differs from previous findings showing preferred navigation of risk carriers along environmental boundaries (*4*, *5*). This discrepancy may be explained by the specific layout of our BPI subtask: The boundary was at considerable distance to potential goal locations, and navigating toward the boundary led to a linear decrease in navigation speed. The exact effects of environmental boundaries on PI performance may be scrutinized in future studies by comparing different types of boundaries (squared and circular) and environmental layouts (distal and/or proximal landmarks).

AD is defined neuropathologically by two hallmarks: neurofibrillary tangles of hyperphosphorylated tau proteins and β-amyloid plaques (*10*, *31*). While neurofibrillary tangles show a sequential spreading from EC to other limbic regions and eventually to multiple areas of the neocortex (*32*), amyloid deposition starts in neocortical areas, followed by a progression toward allocortical brain regions, subcortical structures, the brainstem, and the cerebellum (*31*). The accumulation of MTL tau seems to precede neocortical amyloid deposition in cognitively unimpaired older adults (*33*), although the exact sequence between pathologies and the interpretation to provide when only one pathology is observed is still highly debated. It is generally acknowledged that tau pathology is more closely related to cognitive deficits in preclinical and clinical stages of AD (*33*–*35*). Our and related previous findings in cognitively unimpaired samples at genetic risk for AD showed impaired performance (*5*, *36*) and/or altered strategies (*4*) in navigational tasks that are presumably MTL dependent, whereas performance in navigational tasks that depend primarily on regions outside of the MTL (such as landmark-oriented navigation) was not affected. This performance deficit may thus reflect initial occurrence of AD-related neurofibrillary tangles, rather than early amyloid deposition. However, positron emission tomography (PET) studies of amyloid and tau will be needed to determine the respective contributions of both AD pathologies, and their topographies, to the observed pattern of behavioral performance.

Compensatory strategies of EC-dependent behavioral deficits in *APOE* ε4-carriers may be relevant for the progression of AD pathology: It has been suggested that neural hyperactivity associated with these strategies leads to a progressive deterioration in structural integrity of relevant brain areas (*37*), perhaps by increasing β-amyloid (*38*) and/or tau deposition (*34*). Thus, potential compensatory strategies in risk carriers associated with higher activity in specific brain areas may contribute to AD progression (*4*). Consequently, at later stages of AD, patients should no longer be able to rely on compensatory mechanisms (*39*), leading to worse performance irrespective of whether supportive spatial cues are provided. In a recent study, mild cognitive impairment patients with positive AD biomarkers showed reduced PI performance irrespective of the availability of supportive spatial cues (*36*). At this stage, neural hyperactivity may even directly exert detrimental effects on behavior (*40*). Preventing hyperactivity might thus be a promising therapeutic strategy, possibly improving interneuron dysfunction and counteracting network abnormalities (*41*).

Several limitations of the current study should be addressed. First, our cohort may not be representative of the entire population as the participants were relatively highly educated (average years of education, 13.37 years). Previous studies showed that higher levels of education are associated with a reduced risk of dementia (*42*). Specifically, human PET data indicate a lower impact of tau pathology on neuronal function in AD patients with higher education, suggesting that the level of education might support resilience mechanisms (*43*) [“cognitive reserve hypothesis”; see also (*42*)]. We therefore hypothesize that the *APOE* effects on PI performance we report might actually be more severe in the overall population—a question that should be investigated in future studies.

Another limitation of the current study is that we conducted the fMRI study to reveal the neural mechanisms underlying our specific PI task in a group of participants without any particular impairments due to AD or aging—i.e., in young, healthy participants. Using this approach, we identified different neural processes related to our task. We hypothesize that these physiological neural processes are altered or impaired by age- and/or disease-related factors [e.g., (*4*, *16*, *44*)]. Future studies may thus examine the influence of *APOE* ε4 and age on the physiological neural processes of our PI task.

Last, based on the findings of the current study, we cannot conclude unambiguously that the detrimental effect of *APOE* ε4 on PPI performance results from AD-related pathology. Instead, the selective impairment in PPI and the increased use of landmark-related navigation strategies might constitute an *APOE* ε4–specific variant in neurocognitive development evident even in the healthy state (*45*). Neuropathological studies showed increased AD-related neurofibrillary changes even in young *APOE* ε4-carriers [mean age, 38 years; age range, 22 to 46 years; (*8*)], which may speak in favor of the interpretation that impaired PI performance is a result of very early AD-related changes. Nevertheless, future studies are clearly needed to test for direct relationships between impaired PI performance and signs of AD neuropathology, e.g., using cerebrospinal fluid biomarkers and/or PET imaging.

## CONCLUSION

EC-dependent functioning may prove particularly useful for predicting AD because of its early affection by AD-related neuropathological changes (*10*). Distinct navigational strategies may deteriorate at different preclinical and clinical stages of AD, and PPI may capture the behavioral manifestation of earliest neuropathological changes related to preclinical AD development.

## METHODS

### Participants

We recruited healthy participants at five European sites including Germany (two sites), Spain, Belgium, and Italy (table S1) to (i) examine the influence of *APOE* genotype on PI performance (*APOE* sample), (ii) elucidate the fMRI signatures of PI in our task (fMRI sample), and (iii) investigate associations between brain structure and PI performance (sMRI sample). The study was performed in accordance with the Declaration of Helsinki and was approved by the respective institutional review boards at all sites. All participants gave their written informed consent.

#### APOE sample

Four groups of participants (total, *N* = 318) were recruited at four different sites: Germany (“*APOE* sample 1,” *n* = 112; IfADo—Leibniz Research Centre for Working Environment and Human Factors at the Technical University Dortmund, Dortmund, Germany), Spain (“*APOE* sample 2,” *n* = 114; Pablo de Olavide University, Seville, Spain), Italy (“*APOE* sample 3,” *n* = 68; University of Parma, Parma, Italy), and Belgium (“*APOE* sample 4,” *n* = 24; Cliniques Universitaires Saint-Luc, Brussels, Belgium). Fifty-one participants were excluded because of genotypes other than ε3/ε4 or ε3/ε3 (*n* = 46), prior familiarity with the task (*n* = 1), or technical reasons (*n* = 4), resulting in a final sample of *n* = 267 participants (for demographics, see tables S1 and S2; fig. S1).

#### sMRI sample

The sMRI sample consisted of a subset of participants from *APOE* sample 2 for which sMRI scans were available (*n* = 99; for demographics, see tables S1 and S3; fig. S1).

#### fMRI sample

To elucidate the fMRI signatures of PI in our task, we recruited young healthy participants (*n* = 35; for demographics, see table S1) at Ruhr University Bochum, Bochum, Germany.

#### APOE *genotyping*

Four groups of participants (*APOE* samples 1 to 4) were analyzed for the *APOE* polymorphisms rs429358, a [C/T] substitution on chromosome 19q13.32 of the sequence GCTGGGCGCGGACATGGAGGACGTG[C/T]GCGGCCGCCTGGTGCAGTACCGCGG, and rs7412, a [C/T] substitution of the sequence CCGCGATGCCGATGACCTGCAGAAG[C/T]GCCTGGCAGTGTACCAGGCCGGGGC.

On the basis of the two single-nucleotide polymorphisms (SNPs), participants were assigned to one of the three alleles ε2, ε3, or ε4.

For *APOE* sample 1, venous blood was taken and DNA was isolated using the QIAamp DNA Blood Maxi Kit (Qiagen, Hilden, Germany) according to the manufacturer’s protocol. DNA concentrations were determined using a NanoDrop ND-1000 UV/Vis spectrophotometer (PEQLAB Biotechnologie GMBH, Erlangen, Germany). Genotyping was performed on the ABI7500 Sequence Detection System with the use of TaqMan assays (assay ID: C_3084793_20 for rs429358 and assay ID: C_904973_10 for rs7412; Applied Biosystems, Darmstadt, Germany). Analysis of data was performed according to the manufacturer’s instructions (Applied Biosystems, 7300/7500/7500, Fast Real-Time PCR System Allelic Discrimination Getting Started Guide).

For *APOE* sample 2, genomic DNA was isolated from blood using a standard salting-out protocol. DNA concentration and purity were determined by UV spectrophotometric measurements (Quawell, Q3000 UV). Genotyping was performed by real-time polymerase chain reaction (PCR) (Step-One Plus, Applied Biosystems) using predesigned TaqMan SNP genotyping assays (assay ID: C_3084793_20 for rs429358 and assay ID: C_904973_10 for rs7412; Applied Biosystems, Darmstadt, Germany).

For *APOE* sample 3, genomic DNA was extracted from buccal brushes using the Gentra Puregene Buccal Cell Kit (Qiagen, Valencia, CA, USA). The *APOE* SNPs were genotyped using the ABI PRISM 7700 Sequence Detector (assay ID: C_3084793_20 for rs429358 and assay ID: C_904973_10 for rs7412; Thermo Fisher Scientific, MA, USA) with a TaqMan 5′-allele discrimination Assay-By-Design method (Thermo Fisher Scientific, MA, USA). PCR was performed according to the manufacturer’s instructions.

For *APOE* sample 4, DNA was extracted from blood and analysis of *APOE* polymorphisms was performed by means of restriction enzyme isoform genotyping. We used predesigned TaqMan SNP genotyping assays (assay ID: C_3084793_20 for rs429358 and assay ID: C_904973_10 for rs7412; Applied Biosystems, Darmstadt, Germany).

Participants were not genetically preselected. For analyses, we focused on two genetic subgroups: *APOE* ε3/ε3-carriers [“control group”; common genetic risk for AD (*3*); *n* = 202] and *APOE* ε3/ε4-carriers [“risk group”; increased genetic risk for AD (*3*); *n* = 65]. The prevalence of risk carriers (defined as ε3/ε4 genotypes) in our *APOE* sample is 20.83% (ε3/ε3, 64.74%; ε3/ε4, 20.83%; ε2/ε3, 10.58%; ε2/ε4, 1.60%; ε4/ε4, 1.60%; ε2/ε2, 0.64%). However, please note that the prevalence of the *APOE* ε4 allele in our sample is 12.82% (ε3 allele, 80.45%; ε4 allele, 12.82%; ε2 allele, 6.73%). This relative allele frequency is 50% lower than the prevalence of all ε4 genotypes, because the allele frequency is standardized by the total number of alleles (*N*_allele_ = 624) rather than by the number of subjects/genotypes (*N*_genotype_ = 312). The allele frequency in our sample thus matches previous results with estimates of around 14% (*46*).

Risk group and control group did not differ in terms of demographic characteristics (table S2 for the *APOE* sample and table S3 for the sMRI sample). Participants from other genetic subgroups were excluded as in previous studies (ε2/ε2, *n* = 2; ε2/ε3, *n* = 33; ε2/ε4, *n* = 5; ε4/ε4, *n* = 5) (*4*). Participants and experimenters were blinded toward genotypes. Sample size was based on previous studies, suggesting >50 participants in the smaller genetic subgroup (*7*). All participants reported normal or corrected-to-normal vision and no history of neurological or psychiatric diseases.

### Experimental task

Participants performed a PI task (the Apple Game) in a virtual environment implemented via Unreal Engine (Epic Games, version 4.11). The environment consisted of an endless grassy plane with a blue sky rendered at infinity. Trials were divided into three phases. During the start phase, participants navigated to a goal location, which was marked by a basket (Fig. 1, B and C). Participants were instructed to remember this location. When they had reached the goal location, the basket disappeared and a tree appeared in a different location (outgoing phase). Participants walked toward the tree, checked if it had an apple, and walked to the tree to make it disappear. If the tree did not have an apple, another tree appeared. Trials contained between one and five trees to systematically vary PI difficulty, corresponding to a variation of outgoing distance [Fig. 1C; see also the “Behavioral analyses” section; for similar tasks, see (*16*, *36*)]. The final tree was marked by a red apple (retrieval location), indicating that the participants had to navigate back to the goal location (to place the apple into the basket; incoming phase). After pressing a button to indicate that this was the presumed location of the basket (“response location”), participants received feedback via zero to three stars, depending on the Euclidean distance between the response location and the correct goal location (drop error; Fig. 1D; <1600 vm for three stars, <3200 vm for two stars, and <6400 vm for one star; fig. S1). All phases were self-paced, and only the incoming phase had a time limit of 60 s before the next trial started (this time limit was never reached by any participant). Locations of baskets and trees were equally distributed across an (invisible) grid of 8 × 8 squares (bin edge length, 800 vm) such that each participant visited all squares once in each environmental condition (fig. S1).

Virtual meters are the metric to quantify distances in the virtual environment as given by Unreal Engine. The diameter of the arena was ~13,576 vm (fig. S1A). Therefore, the highest incoming distance of ~7920 vm corresponded to ~58% of the arena diameter. Virtual meters cannot be directly converted into real-world meters, but—assuming that the virtual character’s eye height of 310 vm corresponds to ~1.65 real-world meters—it can be inferred that the virtual arena had a size of ~72 real-world meters.

PI performance was tested in three environmental conditions that differed with regard to the presence or absence of supportive spatial cues. In the PPI condition, the virtual environment did not contain any landmarks or boundaries, and participants purely relied on visual flow to perform PI (Fig. 1A). In the BPI condition, a circular stonewall with a height of 2050 vm surrounded the environment at a radius of 6788 vm (Fig. 1A, middle; for location and radius, see also fig. S1). In the LPI condition, a lighthouse with a height of 1300 vm was present at *x* = 1600 vm and *y* = 800 vm, serving as an intramaze landmark (Fig. 1A; for the location, see also fig. S1). No distal cues outside the boundary were present, which is different from previous studies examining the influence of *APOE* on navigational behavior (*4*, *5*). We chose this design to specifically assess the effects of the boundary and the landmark.

Participants navigated the virtual environments using a joystick (behavioral experiment, Trust GXT 555 Predator; fMRI experiment, MR-compatible joystick from Nata Technologies, Coquitlam, Canada), allowing them to move forward, turn left, or turn right. Moving backward was not possible so that movement direction was equivalent with heading direction. In each subtask, participants’ speed was attenuated when their distance from the center of the arena was larger than 5657 vm and linearly decreased to zero at 6788 vm, ensuring a constant movement radius in subtasks with and without a visible boundary (fig. S1). In this “speed reduction zone,” participants could navigate at full speed when heading toward the center of the arena. The position of the participant was logged every 200 ms, which allowed us to extract movement periods, movement speed, and movement direction.

#### Behavioral experiment

The paradigm was subdivided into subtasks of 16 trials each. Subtasks varied with respect to the layout of the virtual environment. Participants started the first trial of each subtask in the center of the virtual environment (*x* = 0 vm, *y* = 0 vm). The outgoing phase of each subtask contained either one tree (three trials), two trees (three trials), three trees (four trials), four trees (three trials), or five trees (three trials) in randomized order (including the tree marked by an apple). This trial procedure allowed a perfect balancing of locations during the experiment so that each participant visited all 64 squares once in each subtask (fig. S1). Before the beginning of the task, all participants completed eight trials in the PPI condition to practice the paradigm. As we encountered systematic within-subject effects of fixed trial and location sequences in a subgroup of participants (for the exact number of participants, refer to table S2), we fully randomized trials and locations for later participants. The randomization neither showed a main effect on performance (*F* = 0.74, *P* = 0.477) nor did any of the results change when we added randomization version as a covariate to the model (subtask: *F* = 799.09, *P* < 0.001; incoming distance: *F* = 4139.47, *P* < 0.001; “*APOE* × subtask”: *F* = 10.89, *P* < 0.001; performance difference in PPI between risk carriers and controls: *z* = 2.08, *P*_Tukey_ = 0.037). Participants in Parma, Seville, and 20 participants in Brussels completed a long version of the paradigm with six subtasks (2 × PPI, 2 × BPI, and 2 × LPI; Fig. 1E), with the order of the subtasks being pseudorandomized such that the same subtask would not follow each other and the three different subtasks would be equally distributed across the experimental halves. This resulted in a total of 96 experimental trials. Participants could take breaks between the subtasks. The experiment lasted 107.44 ± 33.15 min [mean ± SD)] in total. Participants in Dortmund and four participants in Brussels completed a shorter version of the paradigm with three subtasks (1 × PPI, 1 × BPI, and 1 × LPI; Fig. 1E) with the order of the subtasks being randomized, resulting in 48 experimental trials. Including breaks between the subtasks and practice trials, the short version of the experiment lasted 52.83 ± 12.08 min (mean ± SD) in total.

#### fMRI experiment

The fMRI experiment consisted of a practice run, which was conducted during the structural scan (6 min), and two functional runs. The practice run comprised a maximum of nine trials (three trials in each condition) and ended when the structural scan was over. Each functional run had a duration of 22.32 ± 3.18 min (mean ± SD), which corresponds to 536 ± 76 (mean ± SD) fMRI volumes. The duration of the functional runs varied between participants, because the entire task was self-paced. In between runs, participants could take short breaks. Each run consisted of six blocks containing four trials of each of the three environmental conditions (Fig. 1F), resulting in 16 trials per condition and thus 48 trials in total across the experiment. The order of the blocks was pseudorandomized such that the same subtask would not follow each other and that the three different subtasks would be equally distributed across the two experimental runs. Before every new trial, participants viewed a fixation crosshair with a variable duration of 5 to 7.5 s (randomly distributed).

### Data acquisition

Data were acquired according to a standardized protocol (with regard to experimental setup, instructions, behavior of the investigator, and breaks), ensuring comparability across the five recording sites.

#### Behavioral data acquisition (APOE sample)

The paradigm was presented on laptops with a screen diagonal of 45 cm, a resolution of 1920 × 1080 pixels, and a frame rate of 60 frames/s. We attached the joystick to the table by means of a custom-made frame (identical for all sites), which also served as an armrest, and placed the laptop at a distance of 50 cm in front of the participant.

#### MRI data acquisition (sMRI sample)

Structural brain images were acquired at the Neuroimaging Service of the Pablo de Olavide University (Seville, Spain) using a 3T Philips Ingenia CX MRI scanner equipped with a 32-channel receiver head coil (Philips, Best, The Netherlands). Head motion was minimized by placing foam padding around the subject’s head. One high-resolution three-dimensional (3D) T1-weighted magnetization-prepared rapid gradient echo (MP-RAGE) sequence was acquired in the sagittal plane. Acquisition parameters were empirically optimized to enhance the gray/white matter (WM) contrast with repetition time (TR)/echo time (TE) = 2600/4.7 ms, flip angle (FA) = 9°, voxel resolution = 0.65 mm isotropic, acquisition matrix = 384 mm × 384 mm, resulting in 282 contiguous slices without gap between adjacent slices, acceleration factor (SENSE) = 1.7, and field of view (FOV) = 250 mm × 250 mm × 183 mm.

#### fMRI data acquisition (fMRI sample)

The fMRI recordings were conducted at the Bergmannsheil hospital in Bochum using a 3 T Philips Achieva scanner (Best, The Netherlands) with a 32-channel head coil. High-resolution whole-brain structural brain scans of participants were acquired using a T1-weighted sequence at 1-mm isotropic resolution, an FOV of 240 mm × 240 mm, and 220 transversally oriented slices during a total acquisition time (TA) of 6 min 2 s. Blood oxygenation level–dependent (BOLD) contrast images were measured with a T2*-weighted gradient echo EPI sequence with 2.5-mm isotropic resolution, TR = 2500 ms, TE = 30 ms, FA = 90°, FOV = 96 mm × 96 mm, 46 transversal slices in interleaved order without slice gap, and TA = 22.32 ± 3.18 min (mean ± SD), corresponding to 536 ± 76 volumes (mean ± SD). We discarded the first five images of each session to allow signal steady-state transition. Participants viewed the virtual environment via MR-compatible liquid crystal display (LCD) goggles (VisuaStim Digital, Resonance Technology Inc., Northridge, CA, USA) with a resolution of 800 × 600 pixels, and they navigated the virtual environment by means of an MR-compatible joystick.

### Data analysis

We extracted behavioral data from logfiles using MATLAB (2018a, The MathWorks Inc., Massachusetts) including the Parallel Computing Toolbox (v6.12) and the CircStat Toolbox (*47*). ROIs were created using FreeSurfer (v6.0.0). We used SPM12 (www.fil.ion.ucl.ac.uk/spm) for all fMRI analyses. Statistics were done in R (*48*) (3.5.0) using the lme4 (*49*) (v1.1-17) and emmeans (*40*) (v1.2.2) packages.

#### Behavioral analyses

We determined three different performance measures from the behavioral logfiles (Fig. 1D): drop error, “distance error”, and “rotation error.” The drop error was defined as the Euclidean distance between the goal location and the response location. We computed the distance error as the absolute difference between two distance measures: distance error = *D*_correct_ − *D*_response_, where *D*_correct_ is the distance between the retrieval and the goal location (i.e., the distance of the correct incoming path) and *D*_response_ is the distance between the retrieval and the response location (i.e., the distance of the incoming path chosen by the participant). Rotation error was computed correspondingly as the absolute difference between two angular measures: rotation error = *R*_correct_ − *R*_response_, where *R*_correct_ is the heading direction of the correct incoming path and *R*_response_ is the heading direction of the response path. Only for visualization and enhanced readability, we converted the error measures to performance measures of participant *i* (performance*_i_*) using a linear transformation: performance*_i_* = [max(error) – error*_i_* + min(error)]/max(error) × 1000, where max(error) and min(error) correspond to the maximum and minimum error across all participants, respectively, and error*_i_* corresponds to the error in participant *i*. The formula simply reverses the errors and maps them into the range between 0 and 1000. Note that the linear transformation does not affect the statistical results. Thus, “performance” refers to the drop error, “performance based on distance error” refers to the distance error, and “performance based on rotation error” refers to the rotation error.

To analyze performance as a function of path distance, we calculated incoming and outgoing distance (Fig. 1C): Outgoing distance was determined as the cumulated path distance during the outgoing phase, i.e., we summed the Euclidean distances between “goal,” “tree without apple,” and “retrieval” locations. Incoming distance was defined as the Euclidean distance between the retrieval location and the goal location. Outgoing and incoming distances reflect different subcomponents of PI (keeping track of the traveled path with regard to the goal location and computing a straight line in relation to the goal location, respectively) but are putatively both subject to cumulative error accumulation (*18*).

The precision of PI processes can be improved if sensory information is available to recalibrate the ideothetic coding process (*17*). We thus investigated the influence of spatial cue distance, i.e., the distance between the goal location and the boundary (“goal-to-boundary” distance) or the landmark (“goal-to-landmark” distance) on performance.

Last, we examined navigational strategy, irrespective of performance. To this end, we computed the mean Euclidean distance from the boundary or from the landmark across all time points of the incoming phase (this was not computed during the outgoing phase, because navigation during this period was determined by the consecutive trees).

#### Analysis of everyday life navigational strategies in our samples

We acquired the Santa Barbara Sense of Direction (SBSOD) questionnaire (*51*), which is a self-report measure of navigational abilities in all but one participants of the *APOE* sample (*n* = 266). First, to reveal if *APOE* groups differed with respect to self-reported navigational abilities, we calculated SBSOD mean scores and compared them between risk carriers and controls by means of a *t* test.

Second, to investigate the relationship between *APOE* genotype and navigational strategies, we tested for differences in specific navigational strategy profiles. Specifically, we checked for the proportion of “mappers” and “egocentric navigators” in the *APOE* groups. We defined mappers based on items 7, 9, 13, and 15 in SBSOD:

1) Item 7: I enjoy reading maps.

2) Item 9: I am very good at reading maps.

3) Item 13: I usually let someone else do the navigational planning for long trips. (recoded)

4) Item 15: I don’t have a good “mental map” of my environment. (recoded)

We defined egocentric navigators based on items 4, 6, and 14 in SBSOD:

1) Item 4: My “sense of direction” is very good.

2) Item 6: I very easily get lost in a new city. (recoded)

3) Item 14: I can usually remember a new route after I have traveled it only once.

The mean score of those three items was used to define egocentric navigators. Mappers and egocentric navigators were then classified on the basis of a median split.

#### Statistical analysis

To assess potential demographic differences between risk carriers and controls, we performed two-tailed *t* tests, Wilcoxon tests, and chi-square tests. To test for significant relationships between fMRI representations, we performed bivariate Pearson or Spearman correlations. To disentangle the various factors driving PI performance in the different subtasks of our paradigm, we implemented a mixed linear model using the lme4 package in R (*48*, *49*). We were both interested in fixed effects of predictors varying within subjects and in effects of predictors varying between subjects.

As within-subject predictors, we included subtask (i.e., PPI, LPI, and BPI) and two different types of distance predictors: a path distance predictor that could be quantified in all three subtasks (i.e., outgoing distance or incoming distance), and a spatial cue distance predictor that was only defined in the corresponding subtask (goal-to-boundary distance in the BPI subtask and goal-to-landmark distance in the LPI subtask). In addition, we considered between-subject effects: genotype (i.e., *APOE* ε4-carriers and *APOE* ε4-noncarriers), which was defined for all subjects in the *APOE* sample; volume in EC, HC, or PC/RSC, which was only defined for subjects with available sMRI data (sMRI sample); and representations of integrated path, goal proximity, boundary, landmark, and GLRs, which were only defined for subjects with available fMRI data (fMRI sample).

Continuous variables were centered on the grand mean (between-subject predictors) or on the subject mean (within-subject predictors). We included age and sex as covariates to control for main effects and interactions on subtask, genotype, and gray matter volume. Site and subject were included as random factors. We tested separate models for each outcome variable (performance, performance based on distance error, and performance based on rotation error). Because it is most appropriate for model building and preferable in terms of power (*52*), we always used type II sum of squares (SS) to test fixed effects. For post hoc pairwise comparisons, we used Tukey-adjusted Fisher’s tests as implemented in the emmeans package (*50*). All statistical tests were two-tailed at an alpha level of α < 0.05. We did not report regression coefficients and degrees of freedom, as both are not intuitively interpretable in mixed linear models of high complexity.

We reported all significant main effects and interactions and nonsignificant effects, which were of interest for our hypotheses. Thus, effects that are not reported in the results section were not significant. We did not report significant interactions with covariates (but see fig. S2).

All mixed linear models, except model 6, were built in a hypothesis-driven way (see Table 1 for an overview of all models): Models 1a and 1b were built to test for effects of subtask, genotype, and the two path distance regressors on performance. We used separate models for the two path distance predictors (“a,” outgoing distance; “b,” incoming distance), because they were positively correlated and would have caused multicollinearity. However, to scrutinize whether the two predictors showed significantly different interactions with genotype, we built model 1c. This model contained one regressor that accounted for the effects of the path distance predictors irrespective of the type of path distance and a binary regressor that accounted for the type of path distance (0, outgoing distance; 1, incoming distance).

Models 2a and 2b served to analyze the effects of spatial cue distance, i.e., the subtask-specific measures of the distance between the goal and either the landmark (LPI subtask) or the boundary (BPI subtask). In models 2a and 2b, we analyzed the effects of genotype and goal-to-boundary or goal-to-landmark distance on performance in the respective subtasks.

Models 2c and 2d were the only models in which we did not test for factors influencing PI performance but tested whether *APOE* genotype predicted whether subjects navigated closer to the boundary (BPI subtask) or the landmark (LPI subtask), similar to previous studies (*4*, *5*). Thus, we did not use performance as criterion (i.e., dependent variable) but distance-to-boundary (BPI subtask) or distance-to-landmark (LPI subtask), respectively. These distance measures were obtained by averaging the Euclidean distances across all time points of the incoming phase of each trial of the respective subtasks.

Models 3 to 5 tested for effects of EC, HC, and PC/RSC volumes on PI performance, respectively. These between-subject variables were included in addition to genotype, subtask, and one of the path distance measures. We corrected for multiple comparisons (i.e., the number of ROIs tested) using Bonferroni correction.

We conducted additional analyses to gain a more in-depth and comprehensive understanding of how our genotype effects might vary between different age groups. In detail, we split the sample into two age groups in a data-driven way using MATLAB’s kmeans clustering algorithm. The resulting cutoff was at 42 years, which divided the overall sample into a younger group (*n* = 163; mean age ± SD, 24.32 ± 4.87) and an older group (*n* = 104; mean age ± SD, 58.71 ± 7.75).

The sMRI sample was split up in accordance with the gap in the age histogram (young group: ≤28 years, *n* = 48; mean age ± SD, 22.42 ± 2.28; older group: ≥53 years, *n* = 51; mean age ± SD, 3.22 ± 5.42; fig. S1C). We performed the analyses of models 1 to 3 in the two age groups.

Last, model 6 served to predict performance by means of incoming distance, subtask, and different fMRI representations. As we did not have specific hypotheses about the predictors, this model was built in an exploratory way with the restrictions that overfitting and multicollinearity had to be avoided. The following fMRI representations were considered as model predictors because of significant effects in previous analyses (Figs. 5 and 6):

1) GLRs in bilateral pmEC.

2) Representations of integrated path during outgoing phase in EC.

3) Representations of integrated path during incoming phase in EC.

4) Representations of integrated path during outgoing phase in HC.

5) Representations of integrated path during incoming phase in HC.

6) Representations of goal proximity during outgoing phase in EC.

7) Representations of goal proximity during outgoing phase in HC.

8) Representations of goal proximity during incoming phase in HC.

9) Landmark representations in PC/RSC.

We included (1) in the model as it has been suggested that the grid cell system is particularly involved in PI (*11*). We included (3) in the model because it was significantly correlated with mean drop error across participants (*r*_34_ = −0.541, *P* = 0.001). Because (2), (5), and (7) were correlated with (1) (*r*_34_ = 0.50, *P* = 0.002), (1) (ρ_34_ = −0.38, *P* = 0.024), and (3) (*r*_34_ = −0.341, *P* = 0.045), respectively, they were excluded to avoid multicollinearity. Consequently, we started the model buildup using predictors (1), (3), (4), (6), (8), and (9). These predictors were not correlated (all *r*_34_ or ρ_34_ ≤ 0.32, all *P* ≥ 0.061). We allowed interactions between the fMRI representations and incoming for distance and subtask, but not for interactions between the fMRI representations. In the next step, we aimed at excluding predictors from the model that were not relevant for predicting PI performance to build a parsimonious model (*49*): We started selecting predictors by applying an arbitrary significance threshold of α = 0.1, which resulted in removing (4) and (6). We proceeded with the following procedure: We started with the highest-order interaction term and removed it if it was not significant at α = 0.05. We calculated a new model and successively continued removing lower order terms (as long as they were not part of a higher-order interaction) until there was no nonsignificant term that could be removed. This resulted in a final model including subtask, incoming distance, and (1), (3), (8), (9), as well as their interactions with subtask and incoming distance as predictors (model 6).

#### Creation of ROI masks and sMRI analysis

Anatomical ROIs were created using a semiautomatic approach as implemented in FreeSurfer (https://surfer.nmr.mgh.harvard.edu/). Briefly, sMRIs were processed using the analysis pipeline of FreeSurfer v6.0 that involves intensity normalization, registration to Talairach space, skull stripping, WM segmentation, tessellation of the WM boundary, and automatic correction of topological defects (*53*). Pial surface misplacements and erroneous WM segmentation were manually corrected on a slice-by-slice basis to enhance the reliability of cortical thickness and hippocampal volume measurements. Segmented brain images were parcellated into cortical and subcortical regions (*27*, *54*). This allowed us to obtain volume measurements of HC and EC. For the PC/RSC ROI, we used the posterior-ventral part of the cingulate gyrus derived from the Destrieux atlas [following (*26*), which used this ROI as an approximation for RSC].

Human EC contains structurally and functionally distinct subparts (*28*). Therefore, we subdivided the EC using a template of anterior-lateral EC (alEC) and pmEC based on functional connectivity with these subregions, which represent the human homolog of rodent lateral and medial EC (*28*), respectively. To obtain subject-specific masks, the template was mapped from Montreal Neurological Institute (MNI) space into individual subject’s native space. To ensure that only voxels located in gray matter were analyzed, the ROI masks were thresholded and intersected with a gray matter mask based on FreeSurfer’s cortical parcellation. Each mask was manually inspected to ensure anatomical correctness.

To perform fMRI ROI analyses, ROIs were coregistered to the mean functional image of the respective participant so that all ROI analyses were performed in native space. For the sMRI analysis, we calculated relative gray matter volume by dividing the volume of the ROI by the whole-brain volume of the respective participant (FreeSurfer variable: “BrainSegVolNotVent”).

#### fMRI analysis

##### Preprocessing.

Preprocessing of fMRI data was performed using SPM12 and included slice time correction and spatial realignment. For the whole-brain analysis, we normalized fMRI scans to MNI space using parameters from the normalization procedure of the segmented structural T1 image. Spatial smoothing with a 5-mm isotropic Gaussian kernel was applied to the normalized fMRI data and the fMRI data in native space. Normalized functional images were used to perform whole-brain analyses, while functional images remained in native space for the ROI analyses.

##### General linear models.

Functional images were analyzed via two separate GLMs.

In the PI model (fig. S4), we used four different regressors to model the start phase, the outgoing phase, the incoming phase, and time periods of no movement, separately for each of the three subtasks and each of the two runs (24 regressors). Time periods of no movement were defined as those time periods when movement speed was at zero or minimally above zero (<1 percentile of the subject-specific movement speeds). To include parametric regressors for the moment-to-moment changes in integrated path and goal distance (see below), the regressors for outgoing phase and incoming phase had separate onsets at each movement time point (every 200 ms) and a duration of zero.

We hypothesized that regions involved in PI would represent the integrated path and/or the goal proximity of the current location. We further hypothesized that this representation should be more relevant, and thus more pronounced, when no supportive spatial cues were available (i.e., in the PPI subtask). To test this, we included one of two parametric modulators during the movement periods of the outgoing and incoming phases: integrated path (i.e., the cumulative distance that has been traveled at each time point during the outgoing or incoming phase) or goal distance (i.e., the instantaneous Euclidean distance to the goal during the outgoing or incoming phase).

We assumed that goal proximity and integrated path were correlated, because this is often the case in the real world. To test this assumption empirically, we correlated goal proximity and integrated path across the entire time of the experiment within each participant. We tested each participant’s empirical Spearman ρ value against a surrogate distribution of ρ values established by shuffling the data time series for 10,000 times. We transformed the resulting *P* value into a *z* value. The mean empirical *z* value across participants was tested against a surrogate distribution of mean *z* values established by random sign flips for 10,000 times.

As expected, we found that goal proximity and integrated path were related in our task (ρ = 0.38, *Z* = 3.72, *P* < 0.001). Therefore, we built a third GLM that included both parametric modulators at the same time. Goal proximity was entered first, and integrated path was entered as a second orthogonalized parametric modulator. We then correlated the β values for the integrated path regressor from this GLM with the β values from the GLM that only included the integrated path as parametric modulator to show that representations of integrated path were not a side effect of goal proximity representations.

In the subtask model (fig. S5), we modeled start phase, outgoing phase, incoming phase, and “feedback” separately for each of the three subtasks and each of the two runs (24 regressors). The duration of these regressors corresponded to the duration of the respective phases in each trial. In addition, in the outgoing phase, we included “PI difficulty” as a parametric modulator, reflecting the number of distractor trees in each trial (with values of 1 to 5). This parametric modulator resembles the outgoing distance in the behavioral analyses and was included to reduce error variance in the GLM. We tested two contrasts: “BPI > PPI” and “LPI > PPI”.

All parametric modulators were normalized to values between 0 and 1 and mean centered. All regressors were convolved with the hemodynamic response function before entering the GLM. As nuisance regressors, we included motion parameters as estimated in the realignment procedure and mean value of WM and corticospinal fluid. We did not model temporal derivatives.

##### ROI analysis.

For the ROI analysis, we extracted mean β values (SPM con-files) from our three ROIs (EC, HC, and PC/RSC) in native space and compared them against zero across participants by means of *t* tests for normally distributed data or Wilcoxon’s ranked sum tests otherwise. To compare mean β values across subtasks, we used repeated-measures analysis of variance (ANOVA) or Friedman ANOVA if data did not fulfill normality assumption. To test for differences between two conditions post hoc, we used *t* tests (or Wilcoxon ranked sum tests if normality assumption was violated).

Whenever we used the same model for several ROIs or several contrasts, we corrected for multiple comparisons using FDR, corrected for the number of contrasts and the number of ROIs (α < 0.05) as implemented in R, and adjusted the *P* values accordingly.

For the PI model, we corrected for six multiple comparisons when assessing the relationship between mean β values and the parametric modulators (integrated path or goal proximity, respectively). These six comparisons correspond to three ROIs (EC, HC, and PC/RSC) and two contrasts against zero (one parametric modulator during the outgoing phase and one parametric modulator during the incoming phase).

Only if a contrast tested against zero showed a significant effect for an ROI (corrected for six multiple comparisons), we tested for differences between subtasks (one-way repeated-measures ANOVA with subtask as within-subjects factor) and again corrected for the number of ROIs. We performed two types of post hoc comparisons: (i) We compared the subtasks against each other and corrected for three comparisons (PPI versus BPI, PPI versus LPI, and BPI versus LPI), and (ii) we tested every subtask against zero and likewise corrected for three comparisons (PPI versus 0, BPI versus 0, and LPI versus 0).

For the subtask model, we assessed the influence of either a boundary or a landmark in the environment on BOLD activity and thus corrected for six multiple comparisons (three ROIs: EC, HC, and PC/RSC; two contrasts: BPI > PPI and LPI > PPI). We did not directly compare the boundary and landmark condition.

##### Whole-brain analysis.

In the subtask model, we performed whole-brain analyses on the contrasts between subtasks. Contrast images from the first-level analysis of each participant were entered into a GLM, treating “subjects” as a random effect. Statistical parametric maps were initially thresholded at a family-wise error (FWE)–corrected α level of *P* < 0.05 across the whole brain. We considered clusters significant at *P* < 0.05, FWE-corrected (extent threshold of five voxels). For all significant clusters, we provide maximum probability tissue labels with MNI coordinates derived from the Neuromorphometrics atlas as implemented in SPM12 (www.oasis-brains.org/; http://Neuromorphometrics.com/). Given our hypothesis that EC, HC, and PC/RSC would be involved in our task, we used small volume correction with a mask of the EC, HC, and PC/RSC. We derived these masks from FreeSurfer by warping subject-specific ROIs to standard space, averaging the mask across subjects, and thresholding the composite mask such that a selected voxel was included in the masks of at least 50% of the participants. Thereby, we ensured that results of the ROI analysis and the whole-brain analysis would correspond as closely as possible.

##### Grid-like representations.

To detect GLRs in the fMRI data, we performed a representational similarity analysis following a previous study (*14*). The rationale underlying this analysis is that the activity of a grid cell population should be similar during movements that are n*60° offset from each other (due to the sixfold rotational symmetry of the grid pattern), where *n* = {0, 1, …, 6}. In other words, the activity of a grid cell population should be relatively similar at angular steps of 60°. By contrast, the activity of a grid cell population should be relatively dissimilar during movements that are n*60° + 30° offset from each other. In the following, the former condition is termed “mod(α,60°) = 0°” and the latter condition is termed “mod(α,60°) = 30°” (where α refers to the angular difference between movement directions and “mod” indicates the modulo operator; Fig. 6A).

Analysis steps comprised (i) extracting the voxelwise signal within the ROIs for every fMRI volume; (ii) calculating the mean orientation, speed, and trial number associated with each fMRI volume; (iii) excluding volumes associated with slow movement (<33% average movement speed); (iv) creating means of volumes within bins of 5° orientation based on the information about mean orientation; (v) calculating the angular difference of movement directions between volume bins; (vi) calculating Fisher *z*-transformed Pearson correlations between the volume bins; and (vii) deleting correlations of the same trial to reduce effects of temporal autocorrelations between subsequent fMRI volumes. We compared pattern similarities of angular differences of ±15° from the mod(α,60°) = 0° condition, for which we expected higher pattern similarity, against angular differences of ±15° from the mod(α,60°) = 30° condition, for which we expected lower pattern similarity.

As a control, we re-performed the analysis after excluding correlations based on angular differences of ±15° from 0° to exclude the possibility that GLRs were driven by similarities of activity during movements in the same direction (in 360° space), possibly reflecting a head direction signal. To examine the specificity of sixfold rotational symmetry, we performed further control analyses that tested for other types of rotational symmetry (fourfold, fivefold, sevenfold, and eightfold rotational symmetry).

To exclude possible effects of autocorrelations as a confound, we tested whether the temporal proximity between fMRI volumes assigned to the two conditions was similar. We thus calculated the mean TR difference between every possible combination of angular differences and averaged within the mod(α,60°) = 0° condition and the mod(α,60°) = 30° condition for every participant. We calculated a paired *t* test across participants to ensure that the temporal proximity did not differ between the two conditions. Last, for each participant separately, we checked for uniform sampling of movement directions in 360° and 60° spaces. To this end, we transformed the movement directions (in 360° space or in 60° space) into bins of 5° and tested if the orientation bins deviated from uniformity by means of a Rayleigh test. This resulted in an empirical *z* value that we tested against a surrogate distribution established by shuffling the orientation bins 10,000 times. Comparing the empirical *z* value against the distribution of surrogate *z* values resulted in one *P* value for each participant reflecting how normal or extreme the empirical *z* value of each participant was.

We calculated spatial and temporal signal-to-noise ratios (SNRs) [following (*14*)] by extracting the voxelwise signal of alEC and pmEC for each hemisphere and either calculating the mean and the SD across time points (temporal SNR) or across voxels (spatial SNR). We compared SNRs between EC subregions (alEC versus pmEC) and between hemispheres (left versus right) using a two-way ANOVA. By means of a Pearson correlation, we tested whether the strength of GLRs was significantly related to the SNRs of pmEC.

## Supplementary Material

aba1394_SM.pdf

aba1394_Movie_S1.mp4

## SUPPLEMENTARY MATERIALS

Supplementary material for this article is available at http://advances.sciencemag.org/cgi/content/full/6/35/eaba1394/DC1

## Appendix

View/request a protocol for this paper from *Bio-protocol*.
