# Supplementary material for: Unmasking selective path integration deficits in Alzheimer’s disease risk carriers
Source: Sci Adv. 2020 Aug 28;6(35):eaba1394. doi: 10.1126/sciadv.aba1394 (PMC7455192; doi:10.1126/sciadv.aba1394)
Supplement: aba1394_SM.pdf [file aba1394_SM.pdf]

Science Advances
AAAS

advances.sciencemag.org/cgi/content/full/6/35/eaba1394/DC1

Supplementary Materials for

Unmasking selective path integration deficits in Alzheimer's disease risk carriers

Anne Bierbrauer*, Lukas Kunz*, Carlos A. Gomes, Maike Luhmann, Lorena Deuker, Stephan Getzmann, Edmund Wascher, Patrick D. Gajewski, Jan G. Hengstler, Marina Fernandez-Alvarez, Mercedes Atienza, Davide M. Cammisuli, Francesco Bonatti, Carlo Pruneti, Antonio Percesepe, Youssef Bellaali, Bernard Hanseeuw, Bryan A. Strange, Jose L. Cantero, Nikolai Axmacher*

*Corresponding author. Email: anne.bierbrauer@rub.de (A.B.); lukas.kunz@uniklinik-freiburg.de (L.K.); nikolai.axmacher@rub.de (N.A.)

Published 28 August 2020, *Sci. Adv.* **6**, eaba1394 (2020)
DOI: 10.1126/sciadv.aba1394

The PDF file includes:

Supplementary Text
Figs. S1 to S5
Tables S1 to S5
Legends for movie S1
References

Other Supplementary Material for this manuscript includes the following:

(available at advances.sciencemag.org/cgi/content/full/6/35/eaba1394/DC1)

Movie S1

Supplementary Materials

Supplementary Text

Cognitive status of older participants

We applied the Mini-Mental State Examination in order to assess possible cognitive impairments [MMSE (*23*)]. An MMSE score <24 has been suggested to indicate dementia (*55*), while subjects with MMSE scores ≥24 are considered non-demented. All participants from the "older" group (aged ≥42 years; n = 104) showed MMSE scores of ≥25 (mean ± SD, 28.66 ± 1.33), indicating that all participants in the older sample had normal cognitive abilities. Furthermore, MMSE sum scores in older participants did not differ between genotypes ($t(102) = 0.67$, $P = 0.507$).

All older participants underwent additional cognitive assessments that were specific to each recording site. We used results from these tests to check for mild cognitive impairment in our *APOE* sample (*56*). The criteria for mild cognitive impairment have been defined as a combination of cognitive concerns (subjective memory complaints) and objective evidence for impairment in one or several cognitive domains, typically including memory (*57*). Different cutoffs for cognitive tests have been proposed ranging from -1 standard deviation to -1.5 standard deviation (*57*). All older participants from our sample were above this cutoff, meaning that they did not fulfill the criteria for mild cognitive impairment.

In detail, participants in *APOE* sample 1 (n = 41 older participants) were drawn from a community sample recruited for the "Vital study" (https://www.ifado.de/vital-studie/). Over the course of the "Vital study", participants underwent extensive neuropsychological testing including the German version of the California Verbal Learning Test ["Verbaler Lern- und Merkfähigkeitstest", VLMT (*58*)]. None of the participants performed below -1 standard deviation, indicating that none of the participants would meet the criteria for mild cognitive impairment. In *APOE* sample 2 (n = 51 older participants), all participants were volunteers from aging research programs conducted in the Laboratory of Functional Neuroscience at Pablo de Olavide University (Seville, Spain). All subjects showed a global score of 0 on the Clinical Dementia Rating [CDR (*59*)], which indicates normal cognitive functioning. Subjective memory complaints were excluded by phone interviews with participants and closest relative. *APOE* sample 3 did not contain any participants ≥42 years. In *APOE* sample 4 (n = 1 older participant), participants were recruited through the Memory Clinic of the Neurological Department of the University Clinic Saint-Luc in Brussels. The older participant performed normally on neuropsychological tests including the 10-items verbal memory tests from the CERAD battery (*60*). The participant underwent cognitive testing in the memory clinic because

of subjective memory complaints, but the diagnosis of mild cognitive impairment was excluded by the neurologist.

To conclude, no participant showed mild cognitive impairment or dementia. Subjective memory complaints were not assessed in all participants and their relationship to path integration performance should thus be investigated in future studies.

Experimental and pre-experimental strategies

In principle, the different subtasks could be solved using either implicit or explicit (i.e., declarative) path and time estimation strategies. Specifically, participants could have employed explicit time- or step-counting strategies, which may be less dependent on grid cell-based path integration mechanisms. We largely prevented the use of such strategies by using a joystick (rather than keyboard button presses) as input device. Furthermore, in a post-experimental questionnaire, we asked participants about their navigation strategies. While 24.34% of all participants indicated that they employed some kind of step-counting or time-counting strategies, this proportion did not differ between risk carriers and controls ($\chi^2(1) = 1.14$, $P = 0.320$, table S2), indicating that the effects of *APOE* on path integration performance are not due to alternative path and/or time estimation strategies.

Moreover, *APOE* groups did not differ with respect to self-reported navigational abilities [$t(264) = 0.68$, $P = 0.494$; based on the Santa Barbara Sense of Direction test, ref. (*51*)] or general navigational strategies, i.e., there were similar ratios of “mappers” and “egocentric navigators” among risk carriers and controls (both $\chi^2 \leq 1.29$, both $P \geq 0.308$; table S2). Finally, experience in rural vs. urban navigation should be taken into account in future studies (*61*).

Potential neurophysiological basis of rotation errors

Increased rotation errors might be due to deficits in neural circuits coding for directional information (for example, due to impaired head-direction cells in RSC). However, we would like to point out that (i) grid cells may also be involved in direction coding, (ii) increased rotation errors can directly result from impaired distance coding, for which grid cells have been suggested as the underlying neural substrate, and (iii) RSC engagement did not predict performance in the PPI subtask. Instead, stronger grid-like representations were predictive of better PPI performance.

In support of the first point that grid cells may also provide relevant directional information during navigation, recent simulations have shown that the spike phase of grid cells relative to local field potentials is capable of carrying information about movement direction (*62*). In these simulations,

movement direction could be estimated from the sequence of locations decoded across grid cell phases. It is thus likely that grid cells do not only convey information about translational path integration, but that they are also relevant for the correct estimation of movement directions. Therefore, increased rotation errors during pure path integration in risk carriers as compared to control participants may have directly resulted from impaired grid-cell functioning in our study.

Regarding the second point, rotation errors during the incoming phase can directly result from impaired distance coding during the outgoing phase – and distance coding has been suggested to rely on grid cell functioning [e.g. (*63*)]. The underlying rationale is schematically depicted in figure S3C. For an exemplary trial, we show the correct outgoing path (in black) and the estimated path of this trial (in gray). Here, "estimated path" refers to the subject's mental representation of the correct path, which can be subject to representational errors. In this example, all rotations (angles) have been tracked correctly, and only the translations (distances) have been integrated incorrectly. This incorrect distance coding during the outgoing phase then results in a considerable rotation error during the incoming phase, despite correct angular path integration during the outgoing phase. This example illustrates that errors in translational path integration may induce rotational errors even if angular path integration is perfect. Reduced grid-cell functioning may thus result in impaired distance coding – in turn leading to rotation errors. Indeed, our data show that distance errors and rotation errors correlate significantly ($r(265) = 0.76$, $P < 0.001$), suggesting that they are not independent measures of two distinct functions. Instead, they may reflect the functioning of the same underlying neural substrate (i.e., grid cells).

Finally, with respect to RSC engagement, our mechanistic model (Fig. 7) does not show any influence of RSC activity on performance except via landmark representations in the LPI subtask (Fig. 7D). This speaks against the idea that risk carriers' deficit in the PPI condition is due to RSC malfunctioning. Instead, grid-like representations in the pmEC significantly predicted path integration performance in the PPI subtask (Fig. 7C). Therefore, we argue that the risk carriers' deficit in PPI results from a deficit in grid-like representations. Future fMRI studies will be needed to directly relate path integration deficits of *APOE* risk carriers to impairments in grid-like representations.

Post-hoc analyses of the interaction between EC volume, incoming distance, and APOE

Post-hoc analyses on the interactions between the EC volume, incoming distance, and *APOE* were performed on quintiles of the incoming distance predictor. Since the use of quintiles is an arbitrary

choice, we reanalyzed the post-hoc comparisons using quartiles or tertiles of the incoming distance predictor.

Quartiles: EC volume did not predict performance at any incoming distance in controls (all $z \leq 1.85$, all $P_{\text{Tukey}} \geq 0.124$). We obtained the same result for risk carriers if incoming distances were short (first and second quartile: both $z \leq 1.24$, both $P_{\text{Tukey}} \geq 0.382$). However, at large incoming distances, risk carrier's performance increased significantly with increasing EC volume (third and fourth quartile: both $z \geq 3.01$, both $P_{\text{Tukey}} \leq 0.005$). Directly comparing risk carriers with controls, we found larger effects of EC volume for risk carriers at large incoming distances (fourth quartile: $z = 2.56$, $P_{\text{Tukey}} = 0.036$), but not at shorter incoming distances (first to third quartile: all $z \leq 1.89$, all $P_{\text{Tukey}} \geq 0.176$).

Tertiles: EC volume did not predict performance at any incoming distance in controls (all $z \leq 1.84$, all $P_{\text{Tukey}} \geq 0.128$). For risk carriers, we obtained a significant influence of EC volume for large incoming distances (third tertile: $z = 3.74$, $P_{\text{Tukey}} < 0.001$), but not for shorter incoming distances (first and second tertile: both $z \leq 2.17$, both $P_{\text{Tukey}} \geq 0.058$). Comparing risk carriers with controls, we found no significant difference for the effect of EC volume on performance at lower incoming distances (first and second tertile: both $z \leq 1.33$, both $P_{\text{Tukey}} \geq 0.379$). At high incoming distances, however, EC volume had a more pronounced effect in risk carriers as compared to controls ($z = 2.56$, $P_{\text{Tukey}} = 0.028$).

To conclude, we obtain qualitatively identical results when incoming distance was subdivided into quartiles or tertiles.

Representations of integrated path in EC and HC depend on subtask

During the outgoing phase, EC and HC showed pronounced deactivation with increasing integrated path (both $t_{34} \geq 4.67$, both $P_{\text{FDR}} < 0.001$; Fig. 5A). Deactivation depended on subtask in HC ($F_{2,68} = 4.48$, $P_{\text{FDR}} = 0.030$; Fig. 5C), but not in EC ($F_{2,68} = 1.55$, $P_{\text{FDR}} = 0.221$), and was more pronounced during the PPI and the BPI as compared to the LPI subtask (both $P_{\text{FDR}} \leq 0.026$). HC activity levels were significantly below zero during the PPI and the BPI subtasks (both $t_{34} \geq 4.21$, $P_{\text{FDR}} < 0.001$), but not the LPI subtask ($t_{34} = 0.73$, $P_{\text{FDR}} = 0.471$). By contrast, we observed increased activation in EC and HC in response to integrated path during the incoming phase (both $t_{34} > 2.55$, both $P_{\text{FDR}} < 0.046$; Fig. 5A). These effects did not depend on subtask (both $F_{2,68} \leq 3.23$, both $P_{\text{FDR}} \geq 0.092$; Fig. 5C). Activity in PC/RSC did not show any relationship with integrated path.

During both the outgoing and the incoming phase, HC activity increased with goal proximity (both $t_{34} \geq 2.96$, both $P_{\text{FDR}} \leq 0.028$; Fig. 5B). In EC, we observed this effect only during the outgoing (t_{34}

= 2.86, $P_{FDR} = 0.029$), but not during the incoming phase ($t_{34} = 1.12$, $P_{FDR} = 0.813$). No relationship between goal proximity and PC/RSC activity was observed.

Control analysis of GLMs with integrated path and goal proximity

We tested the neural representation of integrated path and goal proximity in a GLM including both predictors. Goal proximity was entered first and the predictors were orthogonalized. We estimated the beta-values for the model including both predictors at the same time and correlated those beta-values with the beta-values of the individual GLMs. We encountered significant correlations for both predictors, i.e. for goal proximity and for integrated path, and for both phases, i.e. for outgoing and for incoming phase (all r_{34} or $\rho_{34} \geq 0.75$, all $P < 0.001$). This suggests that the neural representation of integrated path in EC and HC is not just a side effect of goal proximity. Instead, the two spatial representations seem to be represented at least partly independently in EC and HC.

Control analyses of GLRs

GLRs were not found in adjacent alEC ($t_{34} = -1.55$, $P = 0.130$), which putatively corresponds to rodent lateral EC (*28*), or in other control ROIs (all $t_{34} \leq 1.04$, all $P \geq 0.307$; Fig. 6C). Furthermore, we did not observe any 4-, 5-, 7- or 8-fold modulation of pattern similarity in bilateral pmEC (all $P \geq 0.169$) or in right pmEC (all $P \geq 0.644$; Fig. 6C). Temporal distances between the two conditions did not differ ($t_{34} = -0.40$, $P = 0.694$), excluding spurious GLRs due to temporal autocorrelations. Rayleigh tests confirmed that movement directions were sampled uniformly in 360° space for all but one participant and in 60° space for all but two participants. Higher spatial and temporal signal-to-noise ratios in pmEC as compared to alEC (both $F_{1,34} \geq 114.90$, both $P < 0.001$) did not correlate with the strength of GLRs (both $|r_{34}| \leq 0.213$; both $P \geq 0.219$), suggesting that selective GLRs in pmEC were not due to higher SNRs in that region.

Supplementary Figures

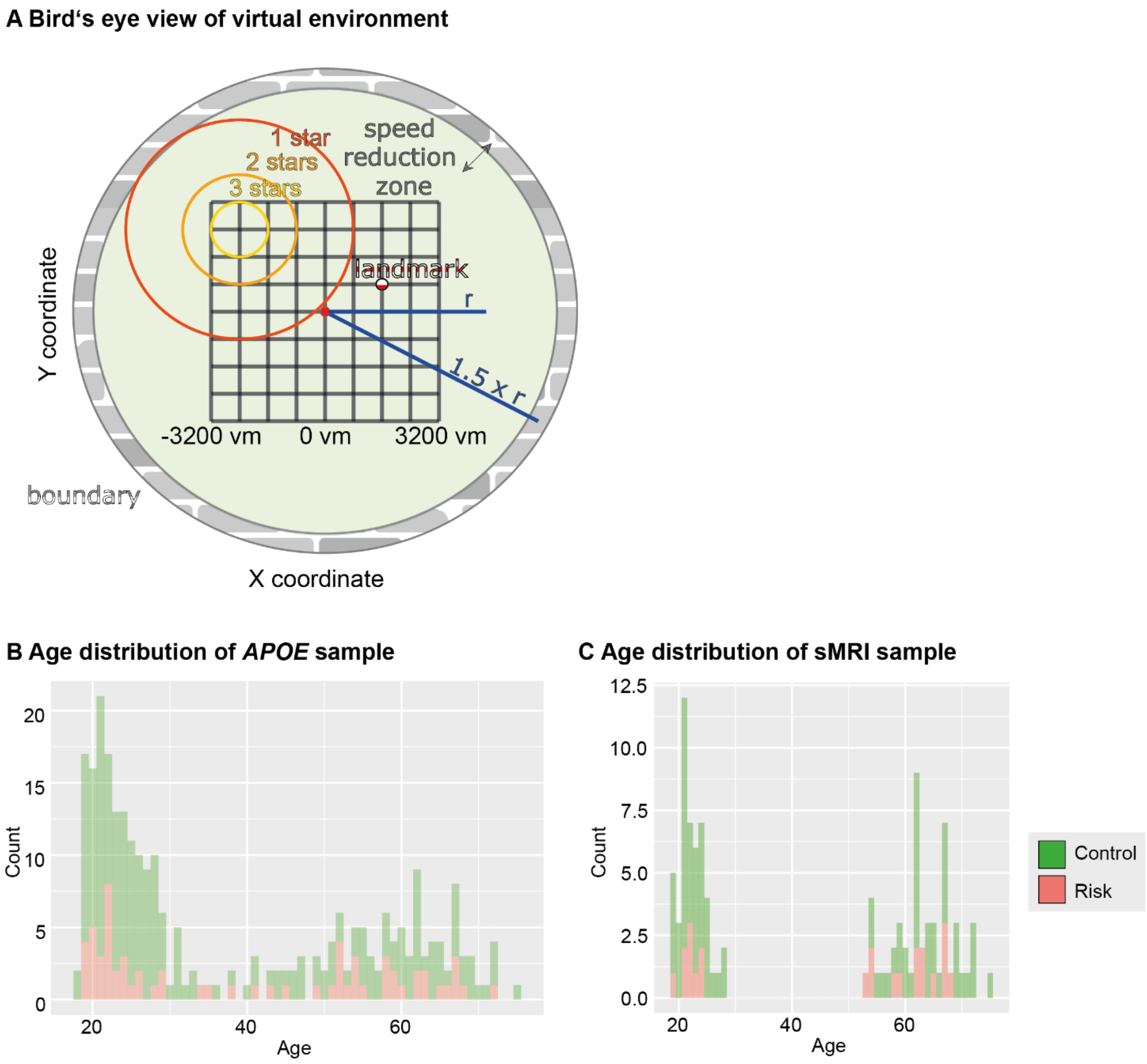

Figure S1. Environmental layout and demographic characteristics of the sample. (**A**) Environmental layout from bird's eye view. Locations of baskets (i.e., goal locations) and trees were equally distributed across a grid of 8x8 squares such that each participant visited all squares once in each subtask (to ensure good coverage of the entire virtual environment). Feedback was given according to the Euclidean distance between the response location and the correct goal location (i.e., drop error). In all subtasks, participants' speed was linearly decreased to zero when their distance from the center of the arena was larger than 1.25*r vm. In BPI, a circular boundary surrounded the environment at a distance of 1.5*r vm. In LPI, a landmark was located close to the center of the environment (at x = 1600 vm, y = 800 vm). (**B**) Age distribution of the *APOE* sample; (**C**) Age distribution of the sMRI sample. BPI, boundary-supported path integration; LPI, landmark-supported path integration; r, radius; vm, virtual meters, Control, *APOE* ε3/ε3-carriers; risk, *APOE* ε3/ε4-carriers.

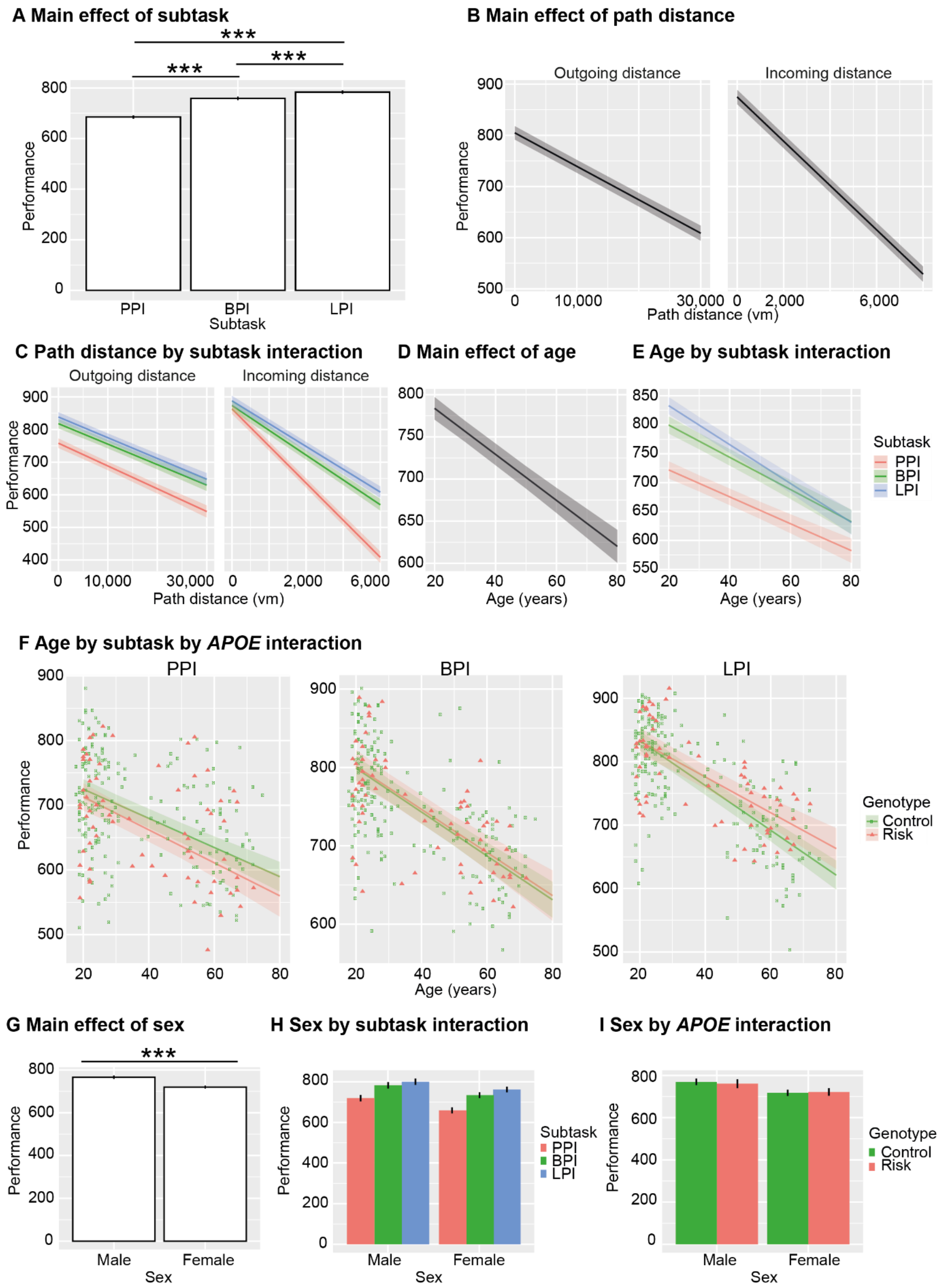

Figure S2. Effects of subtask, path distance, age, and sex on PI performance. (**A**) Main effect of subtask. Performance was better in LPI than in BPI and better in BPI than in LPI. (**B**) Main effects of outgoing (model 1a) and incoming (model 1b) distance. Performance was better at lower outgoing and lower incoming distances. (**C**) No interaction of subtask by outgoing distance (model 1a), but significant interaction of subtask by incoming distance (model 1b). Incoming distance had a more pronounced effect in the PPI subtask than in the two other subtasks. (**D**) Main effect of age: Younger participants performed better (both $F > 262.40$, both $P < 0.001$). (**E**) Age by subtask interaction ($F = 28.96$, $P < 0.001$). Performance in the LPI subtask deteriorated more strongly with older age as compared to the other subtasks (both $z > 2.65$, both $P_{\text{Tukey}} < 0.022$), with no difference between PPI and BPI ($z = 2.15$, $P_{\text{Tukey}} = 0.081$). This result is in line with a recent publication showing that older age is associated with impaired landmark navigation

(*44*). (**F**) Significant age by subtask by *APOE* interaction ($F = 5.79$, $P = 0.003$), but risk carriers did not differ from controls with respect to age-related decline of performance in any of the subtasks (all $z < 1.79$, all $P_{\text{Tukey}} = 0.171$). (**G**) Main effect of sex: Male participants performed better than females (both $F > 63.49$, both $P < 0.001$). (**H**) Significant sex by subtask interaction ($F = 9.38$, $P < 0.001$). Both sexes showed significant differences between the subtasks (all $z \geq 4.06$, all $P < 0.001$), but females benefited more from boundaries and landmarks as indicated by a significantly larger performance increase in the BPI ($z = 2.72$, $P = 0.020$) and in the LPI ($z = 4.72$, $P < 0.001$) as compared to the PPI subtask. (**I**) Significant sex by *APOE* interaction ($F = 3.90$, $P = 0.049$), but risk carriers did not differ from controls with respect to sex-related differences in performance (both $z < 1.50$, both $P_{\text{Tukey}} > 0.220$). (**A**, **D**, **E**, **F**, **G**, **H**, **I**) depict results for model 1b. Results for model 1a are statistically equivalent. Error bars (**A**, **G**, **H**, **I**), s.e.m.; shaded areas (**B**, **C**, **D**, **E**, **F**), s.e.m.; every dot in (**F**) reflects the data of one participant; ***$P < 0.001$; PI, path integration; PPI, pure path integration; BPI, boundary-supported path integration; LPI, landmark-supported path integration; Control, *APOE* ε3/ε3-carriers; Risk, *APOE* ε3/ε4-carriers; vm, virtual meters.

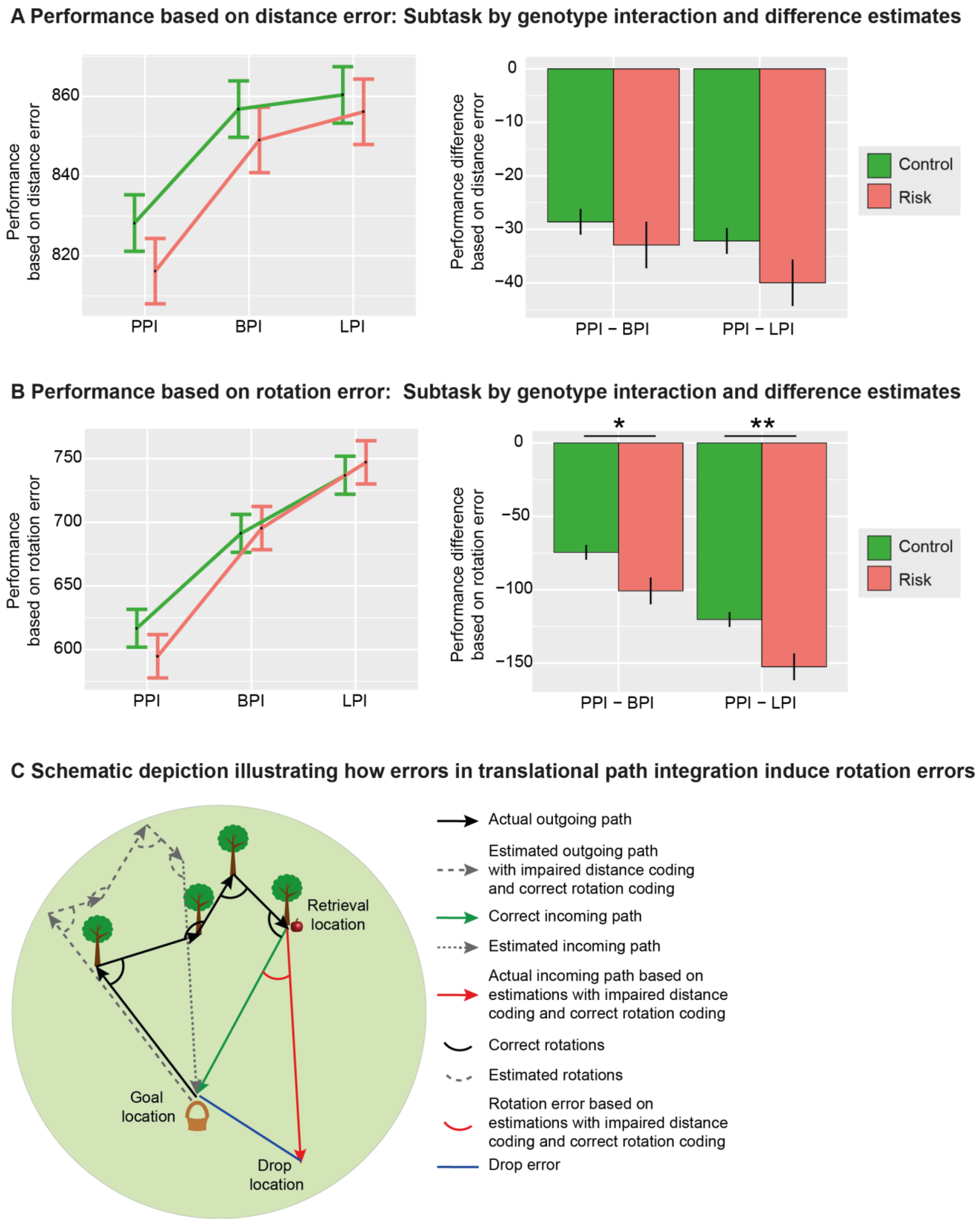

Figure S3. Performance based on distance and rotation error. (**A**) Performance based on the distance error showed no significant genotype by subtask interaction (both $F < 0.95$, left) and no significant improvements due to supportive spatial cues (right). (**B**) Performance based on rotation error showed a significant genotype by subtask interaction (both $F > 8.65$, left) and significant improvements due to supportive spatial cues (both $P < 0.031$, right). (**C**) Errors in translational path integration induce rotation errors even when angular path integration is perfect. (**A**, **B**) depict results for model 1b and results for model 1a are statistically equivalent. Y-axes show parameter estimates; error bars (**A**, **B**), s.e.m.; $^*P < 0.05$; $^{**}P < 0.01$; Control, *APOE* ε3/ε3-carriers; Risk, *APOE* ε3/ε4-carriers; PPI, pure path integration; BPI, boundary-supported path integration; LPI, landmark-supported path integration.

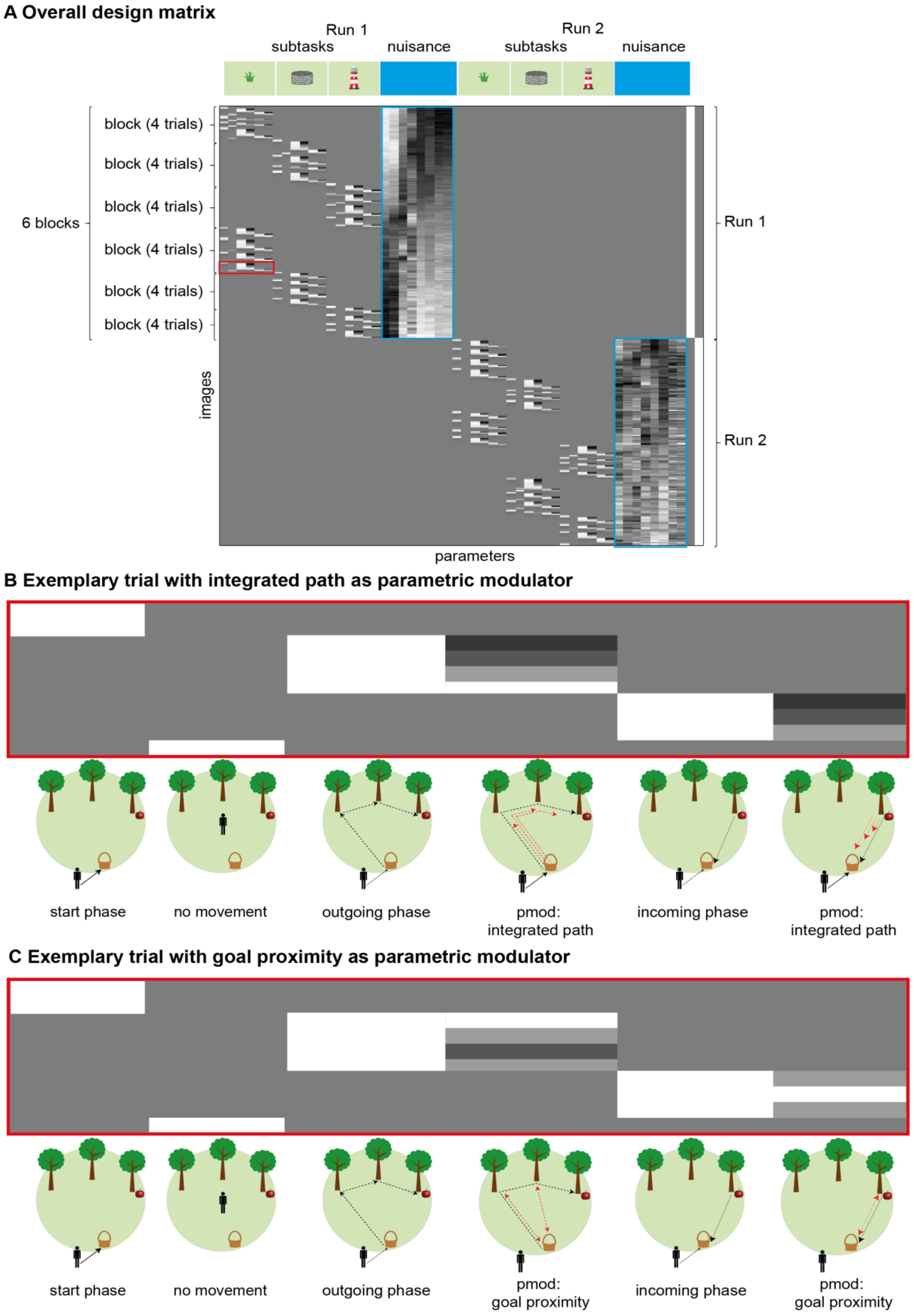

Figure S4. Exemplary design matrix of the PI model. (**A**) Overall design matrix for one participant. (**B**) Start phase, phases of no movement, outgoing phase, and incoming phase were modeled separately for each subtask and run. In this exemplary trial, the participant moves during the entire outgoing and incoming phase, and stops moving during feedback at the end of the trial. Onsets of these regressors were modeled at the sampling rate of the behavioral data (5 Hz temporal resolution). Here, integrated path, which is the cumulated path distance until that time point, was modeled as one of two possible parametric modulators. (**C**) Same as (**B**),

but with goal proximity, which is the inverted Euclidean distance to the goal at that time point, as parametric modulator. (**B**) and (**C**) show a magnification of the red box in (**A**). pmod, parametric modulator.

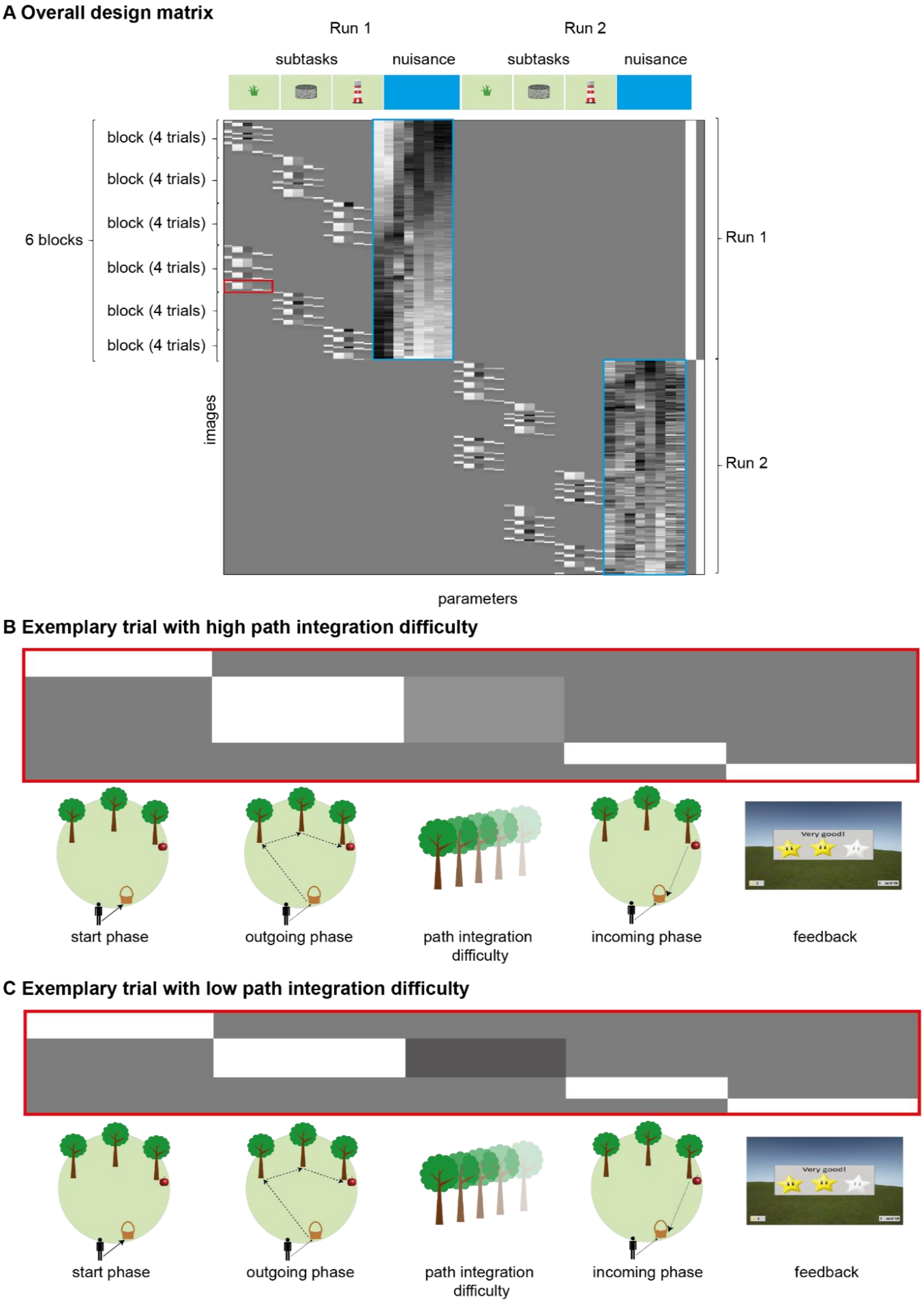

Figure S5. Exemplary design matrix of the subtask model. (**A**) Overall design matrix. (**B**) Start phase, outgoing phase, incoming phase, and feedback were modeled separately for each subtask and run. Onsets of the regressors correspond to the start of the respective phase; durations of the regressors depend on the duration of the respective phase. In the outgoing phase, a parametric modulator (PI difficulty) was modeled, varying between 1 and 5 depending on the number of trees in the respective trial. In this exemplary trial, PI difficulty was high. (**C**) Same as (**B**), but in this exemplary trial, PI difficulty was low. (**B**) and (**C**) show a magnification of the red box in (**A**).

Supplementary Tables

Table S1. Sample information.

Sample	Site	Number of participants [male/female]	Mean age (± s.d.), age range (years)	Genotypes ε3/ε3 vs. ε3/ε4	Structural MR available
fMRI sample	Ruhr-University Bochum, Bochum, Germany	35 [17/18]	24.97 (± 3.98), 19-35		
APOE sample 1	IfADo – Leibniz Research Centre for Working Environment and Human Factors at the Technical University Dortmund, Dortmund, Germany	87 [25/62]	44.20 (± 13.60), 21-68	64 vs. 23	
APOE sample 2; sMRI sample	Pablo de Olavide University, Seville, Spain	99 [48/51]	43.43 (± 20.92), 19-75	76 vs. 23	99
APOE sample 3	University of Parma, Parma, Italy	62 [21/41]	22.42 (± 4.10), 18-38	49 vs. 13	
APOE sample 4	Cliniques Universitaires Saint-Luc, Université Catholique de Louvain, Brussels, Belgium	19 [9/10]	28.16 (± 11.00), 22-72	13 vs. 6	
APOE sample Total	Dortmund, Seville, Parma, Brussels	267 [103/164]	37.72 (± 17.89), 18-75	202 vs. 65	99

Values denote mean (± s.d.) or the number of participants.

Table S2. Demographic and experiment characteristics of the *APOE* sample.

	Control group (*APOE* ε3/ε3)	Risk group (*APOE* ε3/ε4)	*P*
Number	202	65	
Demographic characteristics			
Age (± s.e.m.), age range [years]	37.06 (± 1.25), 18-75	39.75 (± 2.25), 19-72	0.545[c] 0.287[d]
Sex [male/female]	80/122	23/42	0.543[b]
Years of education (± s.e.m.) [years]	13.78 (± 0.24)	13.58 (± 0.41)	0.495[c]
MMSE score (± s.e.m.) of participants ≥42 years	28.61 (± 0.16)	28.80 (± 0.21)	0.507[a]
SBSOD score (± s.e.m.)	3.48 (± 0.07)	3.57 (± 0.11)	0.494[a]
Mappers [yes/no]	101/201	32/65	1.000[b]
Egocentric navigators [yes/no]	84/201	22/65	0.308[b]
Site [Dortmund/Seville/Parma/Brussels]	64/49/76/13	23/13/23/6	0.754[b]
Experiment characteristics			
Version [Fixed trial sequence/Random trial sequence/Random locations]	24/29/149	4/13/48	0.282[b]
Speed [vm/s]	626.91 (± 5.90)	620.54 (± 10.11)	0.664[c]
Rotation speed [degrees/s]	94.76 (± 1.99)	90.47 (± 3.73)	0.188[c]
Experiment duration [min]	89.68 (± 2.74)	87.58 (± 4.37)	0.914[c]
Subtask duration: PPI [min]	15.95 (± 0.48)	15.59 (± 0.59)	0.641[c]
Subtask duration: BPI [min]	15.40 (± 0.36)	15.49 (± 0.53)	0.472[c]
Subtask duration: LPI [min]	16.10 (± 0.33)	15.85 (± 0.55)	0.823[c]
Practice duration [min]	9.20 (± 0.30)	9.45 (± 0.45)	0.272[c]
Break duration [min]	9.57 (± 0.78)	8.81 (± 1.30)	0.835[c]
Time/Step counting strategies [yes/no]	46/156	19/46	0.320[b]

Values denote mean (± s.e.m.) or the number of participants. *P*-values refer to (a) two-sample *t*-tests, (b) χ^2-tests, (c) Mann Whitney *U*-tests, (d) χ^2-test across age quintiles. MMSE, Mini-Mental State Examination; SBSOD, Santa Barbara Sense of Direction; PPI, pure path integration; BPI, boundary-supported path integration; LPI, landmark-supported path integration; vm, virtual meters.

Table S3. Demographic and experiment characteristics of the sMRI sample.

	Control group (*APOE* ε3/ε3)	Risk group (*APOE* ε3/ε4)	*P*
Number	76	23	
Demographic characteristics			
Age (± s.e.m.), age range [years]	42.63 (± 2.43)<br>[19-75]	46.09 (± 4.21)<br>[19-68]	0.724[c]<br>0.870[d]
Sex [male/female]	36/40	12/11	0.686[b]
Years of education [years]	12.24 (± 0.41)	11.43 (± 0.68)	0.237[c]
MMSE score (± s.e.m.) of participants ≥42 years	28.89 (± 0.21)	28.71 (± 0.30)	0.648[a]
Experiment characteristics			
Version [Fixed trial sequence/Random trial sequence/Random locations]	0/9/67	0/1/22	0.296[b]
Speed [vm/s]	641.79 (± 7.40)	636.22 (± 12.82)	0.999[c]
Rotation speed [degrees/s]	101.81 (± 3.04)	104.74 (± 7.65)	0.807[c]
Experiment duration [min]	123.02 (± 3.90)	117.02 (± 6.99)	0.458[c]
Subtask duration: PPI [min]	19.05 (± 0.70)	17.56 (± 1.34)	0.206[c]
Subtask duration: BPI [min]	18.22 (± 0.66)	17.68 (± 1.10)	0.706[c]
Subtask duration: LPI [min]	18.97 (± 0.60)	18.37 (± 1.09)	0.640[c]
Practice duration [min]	10.84 (± 0.61)	9.76 (± 0.86)	0.548[c]
Break duration [min]	20.43 (± 1.24)	19.58 (± 2.25)	0.646[c]
Gray matter volume			
Relative bilateral EC volume [% of whole brain volume]	0.26 (± 0.004)	0.27 (± 0.09)	0.231[a]
Relative bilateral HC volume [% of whole brain volume]	0.69 (± 0.006)	0.71 (± 0.013)	0.236[c]
Relative bilateral PC/RSC volume [% of whole brain volume]	0.12 (± 0.002)	0.12 (± 0.002)	0.121[a]
Brain volume (BrainSegVolNotVent) [mm^3]	1,094,888.75<br>(± 13,150.44)	1,065,430.39<br>(± 25,786.52)	0.267[c]

Values denote mean (± s.e.m.) or the number of participants. *P*-values refer to (a) two-sample *t*-tests, (b) χ^2-tests, (c) Mann Whitney *U*-tests, (d) Fisher's exact test across age quintiles (due to the occurrence of expected values < 5); MMSE, Mini-Mental State Examination; PPI, pure path integration; BPI, boundary-supported path integration; LPI, landmark-supported path integration; vm, virtual meters; EC, entorhinal cortex; HC, hippocampus; PC/RSC, retrosplenial cortex.

Table S4. Behavioral results of *APOE* sample split by age groups.

Model	Effect	*APOE* sample	*APOE* sample Younger (<42 years)	*APOE* sample Older (≥42 years)
**1a**	Subtask	*	*	*
	Outgoing distance	*	*	*
	Subtask by outgoing distance	-	-	-
	Gender	*	*	*
	Age	*	*	*
	APOE	-	-	-
	APOE by subtask	*	*	*
	APOE by outgoing distance	-	-	-
	risk vs. control: PPI	*	-	*
**1b**	Subtask	*	*	*
	Incoming distance	*	*	*
	Subtask by incoming distance	*	*	*
	Gender	*	*	*
	Age	*	*	*
	APOE	-	-	-
	APOE by subtask	*	*	*
	APOE by incoming distance	*	-	-
	risk vs. control: PPI	*	-	*
**2a**	Goal-to-boundary distance	*	*	*
	APOE by goal-to-boundary distance	-	*	-
**2b**	Goal-to-landmark distance	*	*	*
	APOE by goal-to-landmark distance	*	-	-
**2c**	*APOE*	-	-	-
**2d**	*APOE*	*	*	+

* significant: $P < 0.050$; + trend: $0.050 \leq P < 0.100$; - not significant; PPI, pure path integration.

Table S5. Global and local maxima of whole brain analysis for "Subtask" contrasts.

Brain regions exhibiting BOLD activations or deactivations during BPI or LPI as compared to the PPI subtask. Reported are all clusters located in gray matter with more than 5 voxels, surviving an initial height threshold of $P < 0.05$, FWE-corrected for whole brain, and a cluster-level FWE correction at $P < 0.05$, as well as small volume corrected (SVC) clusters for EC, HC, and PC/RSC. Clusters within ROIs are marked *italic*. Clusters depicted in Fig. 3 are marked with a *. For other significant clusters, maximum probability tissue labels are derived from the Neuromorphometrics atlas contained in SPM. L, left; R, right.

Region	Voxels	MNI coordinates			Z-score
		X	Y	Z	
BPI > PPI					
R lingual gyrus*	991	6	-75	0	>15
R PC/RSC (SVC)	10	8	-52	5	4.33
PPI > BPI	*No significant clusters*				
LPI > PPI					
L precuneus	119	-2	-57	50	6.74
L middle occipital gyrus	79	-50	-72	23	5.94
R middle occipital gyrus	38	41	-77	35	5.95
R middle occipital gyrus	44	46	-72	20	5.92
R lingual gyrus	7	28	-40	-10	5.69
R thalamus	9	21	-30	5	5.59
L precuneus	37	-15	-67	30	5.57
R precuneus	17	18	-65	28	5.56
R cerebellum	9	11	-60	-53	5.36
L occipital fusiform gyrus	5	-22	70	-8	5.07
R PC/RSC (SVC)*	28	8	-47	5	5.15
L PC/RSC (SVC)	5	-7	-47	3	3.86
PPI > LPI	*No significant clusters*				

Supplementary Movies

Movie S1. Paradigm.

REFERENCES AND NOTES

1. G. Coughlan, J. Laczó, J. Hort, A.-M. Minihane, M. Hornberger, Spatial navigation deficits—Overlooked cognitive marker for preclinical Alzheimer disease? *Nat. Rev. Neurol.* **14**, 496–506 (2018).
2. R. A. Sperling, C. R. Jack Jr., P. S. Aisen, Testing the right target and right drug at the right stage. *Sci. Transl. Med.* **3**, 111cm33 (2011).
3. E. H. Corder, A. M. Saunders, W. J. Strittmatter, D. E. Schmechel, P. C. Gaskell, G. W. Small, A. D. Roses, J. L. Haines, M. A. Pericak-Vance, Gene dose of apolipoprotein E type 4 allele and the risk of Alzheimer's disease in late onset families. *Science* **261**, 921–923 (1993).
4. L. Kunz, T. N. Schröder, H. Lee, C. Montag, B. Lachmann, R. Sariyska, M. Reuter, R. Stirnberg, T. Stöcker, P. C. Messing-Floeter, J. Fell, C. F. Doeller, N. Axmacher, Reduced grid-cell–like representations in adults at genetic risk for Alzheimer's disease. *Science* **350**, 430–433 (2015).
5. G. Coughlan, A. Coutrot, M. Khondoker, A.-M. Minihane, H. Spiers, M. Hornberger, Toward personalized cognitive diagnostics of at-genetic-risk Alzheimer's disease. *Proc. Natl. Acad. Sci. U.S.A.* **116**, 9285–9292 (2019).
6. C. R. A. Mondadori, D. J.-F. de Quervain, A. Buchmann, H. Mustovic, M A. Wollmer, C. F. Schmidt, P. Boesiger, C. Hock, R. M. Nitsch, A. Papassotiropoulos, K. Henke, Better memory and neural efficiency in young apolipoprotein E ε4 carriers. *Cereb. Cortex* **17**, 1934–1947 (2007).
7. G. H. Weissberger, D. A. Nation, C. P. Nguyen, M. W. Bondi, S. D. Han, Meta-analysis of cognitive ability differences by apolipoprotein E genotype in young humans. *Neurosci. Biobehav. Rev.* **94**, 49–58 (2018).
8. E. Ghebremedhin, C. Schultz, E. Braak, H. Braak, High frequency of apolipoprotein E ϵ4 allele in young individuals with very mild Alzheimer's disease-related neurofibrillary changes. *Exp. Neurol.* **153**, 152–155 (1998).
9. B. J. Hanseeuw, R. A. Betensky, H. I. L. Jacobs, A. P. Schultz, J. Sepulcre, J A. Becker, D. M. O. Cosio, M. Farrell, Y. T. Quiroz, E. C. Mormino, R. F. Buckley, K. V. Papp, R. A. Amariglio, I. Dewachter, A. Ivanoiu, W. Huijbers, T. Hedden, G. A. Marshall, J. P. Chhatwal, D. M. Rentz, R. A. Sperling, K. Johnson, Association of amyloid and tau with

cognition in preclinical Alzheimer disease: A longitudinal study. *JAMA Neurol.* **76**, 915–924 (2019).

10. H. Braak, D. R. Thal, E. Ghebremedhin, K. Del Tredici, Stages of the pathologic process in Alzheimer disease: Age categories from 1 to 100 years. *J. Neuropathol. Exp. Neurol.* **70**, 960–969 (2011).
11. T. Hafting, M. Fyhn, S. Molden, M.-B. Moser, E. I. Moser, Microstructure of a spatial map in the entorhinal cortex. *Nature* **436**, 801–806 (2005).
12. H. Fu, G. A. Rodriguez, M. Herman, S. Emrani, E. Nahmani, G. Barrett, H. Y. Figueroa, E. Goldberg, S. A. Hussaini, K. E. Duff, Tau pathology induces excitatory neuron loss, grid cell dysfunction, and spatial memory deficits reminiscent of early Alzheimer's disease. *Neuron* **93**, 533–541.e5 (2017).
13. C. F. Doeller, C. Barry, N. Burgess, Evidence for grid cells in a human memory network. *Nature* **463**, 657–661 (2010).
14. J. L. S. Bellmund, L. Deuker, T. N. Schröder, C. F. Doeller, Grid-cell representations in mental simulation. *eLife* **5**, e17089 (2016).
15. L. Kunz, S. Maidenbaum, D. Chen, L. Wang, J. Jacobs, N. Axmacher, Mesoscopic neural representations in spatial navigation. *Trend Cogn. Sci.* **23**, 615–630 (2019).
16. M. Stangl, J. Achtzehn, K. Huber, C. Dietrich, C. Tempelmann, T. Wolbers, Compromised grid-cell-like representations in old age as a key mechanism to explain age-related navigational deficits. *Curr. Biol.* **28**, 1108–1115.e6 (2018).
17. K. Hardcastle, S. Ganguli, L. M. Giocomo, Environmental boundaries as an error correction mechanism for grid cells. *Neuron* **86**, 827–839 (2015).
18. Y. Burak, I. R. Fiete, Accurate path integration in continuous attractor network models of grid cells. *PLOS Comput. Biol.* **5**, e1000291 (2009).
19. M. Gil, M. Ancau, M. I. Schlesiger, A. Neitz, K. Allen, R. J. De Marco, H. Monyer, Impaired path integration in mice with disrupted grid cell firing. *Nat. Neurosci.* **21**, 81–91 (2018).
20. L. R. Howard, A. H. Javadi, Y. Yu, R. D. Mill, L. C. Morrison, R. Knight, M. M. Loftus, L. Staskute, H. J. Spiers, The hippocampus and entorhinal cortex encode the path and Euclidean distances to goals during navigation. *Curr. Biol.* **24**, 1331–1340 (2014).
21. R. A. Epstein, E. Z. Patai, J. B. Julian, H. J. Spiers, The cognitive map in humans: Spatial navigation and beyond. *Nat. Neurosci.* **20**, 1504–1513 (2017).

22. A. S. Mitchell, R. Czajkowski, N. Zhang, K. Jeffery, A. J. D. Nelson, Retrosplenial cortex and its role in spatial cognition. *Brain Neurosci. Adv.* **2**, 2398212818757098 (2018).
23. M. F. Folstein, S. E. Folstein, P. R. McHugh, "Mini-mental state". A practical method for grading the cognitive state of patients for the clinician. *J. Psychiatr. Res.* **12**, 189–198 (1975).
24. J. Miller, A. J. Watrous, M. Tsitsiklis, S. A. Lee, S. A. Sheth, C. A. Schevon, E. H. Smith, M. R. Sperling, A. Sharan, A. A. Asadi-Pooya, G. A. Worrell, S. Meisenhelter, C. S. Inman, K. A. Davis, B. Lega, P. A. Wanda, S. R. Das, J. M. Stein, R. Gorniak, J. Jacobs, Lateralized hippocampal oscillations underlie distinct aspects of human spatial memory and navigation. *Nat. Commun.* **9**, 2423 (2018).
25. S. Matura, D. Prvulovic, A. Jurcoane, D. Hartmann, J. Miller, M. Scheibe, L. O'Dwyer, V. Oertel-Knöchel, C. Knöchel, B. Reinke, T. Karakaya, F. Fußer, J. Pantel, Differential effects of the ApoE4 genotype on brain structure and function. *Neuroimage* **89**, 81–91 (2014).
26. J. P. Shine, J. P. Valdés-Herrera, M. Hegarty, T. Wolbers, The human retrosplenial cortex and thalamus code head direction in a global reference frame. *J. Neurosci.* **36**, 6371–6381 (2016).
27. C. Destrieux, B. Fischl, A. Dale, E. Halgren, Automatic parcellation of human cortical gyri and sulci using standard anatomical nomenclature. *Neuroimage* **53**, 1–15 (2010).
28. T. Navarro Schröder, K. V. Haak, N. I. Z. Jimenez, C. F. Beckmann, C. F. Doeller, Functional topography of the human entorhinal cortex. *eLife* **4**, e06738 (2015).
29. A. Coutrot, R. Silva, E. Manley, W. de Cothi, S. Sami, V. D. Bohbot, J. M. Wiener, C. Hölscher, R. C. Dalton, M. Hornberger, H. J. Spiers, Global determinants of navigation ability. *Curr. Biol.* **28**, 2861–2866.e4 (2018).
30. S. A. Marchette, L. K. Vass, J. Ryan, R. A. Epstein, Anchoring the neural compass: Coding of local spatial reference frames in human medial parietal lobe. *Nat. Neurosci.* **17**, 1598–1606 (2014).
31. D. R. Thal, U. Rüb, M. Orantes, H. Braak, Phases of Aβ-deposition in the human brain and its relevance for the development of AD. *Neurology* **58**, 1791–1800 (2002).
32. H. Braak, I. Alafuzoff, T. Arzberger, H. Kretzschmar, K. Del Tredici, Staging of Alzheimer disease-associated neurofibrillary pathology using paraffin sections and immunocytochemistry. *Acta Neuropathol.* **112**, 389–404 (2006).

33. W. Jagust, Imaging the evolution and pathophysiology of Alzheimer disease. *Nat. Rev. Neurosci.* **19**, 687–700 (2018).
34. W. Huijbers, A. P. Schultz, K. V. Papp, M. R. LaPoint, B. Hanseeuw, J. P. Chhatwal, T. Hedden, K. A. Johnson, R. A. Sperling, Tau accumulation in clinically normal older adults is associated with hippocampal hyperactivity. *J. Neurosci.* **39**, 548–556 (2019).
35. A. Bejanin, D. R. Schonhaut, R. La Joie, J. H. Kramer, S. L. Baker, N. Sosa, N. Ayakta, A. Cantwell, M. Janabi, M. Lauriola, J. P. O'Neil, M. L. Gorno-Tempini, Z. A. Miller, H. J. Rosen, B. L. Miller, W. J. Jagust, G. D. Rabinovici, Tau pathology and neurodegeneration contribute to cognitive impairment in Alzheimer's disease. *Brain* **140**, 3286–3300 (2017).
36. D. Howett, A. Castegnaro, K. Krzywicka, J. Hagman, D. Marchment, R. Henson, M. Rio, J. A. King, N. Burgess, D. Chan, Differentiation of mild cognitive impairment using an entorhinal cortex-based test of virtual reality navigation. *Brain* **142**, 1751–1766 (2019).
37. M. Ewers, R. A. Sperling, W. E. Klunk, M. W. Weiner, H. Hampel, Neuroimaging markers for the prediction and early diagnosis of Alzheimer's disease dementia. *Trends Neurosci.* **34**, 430–442 (2011).
38. W. J. Jagust, E. C. Mormino, Lifespan brain activity, β-amyloid, and Alzheimer's disease. *Trends Cogn. Sci.* **15**, 520–526 (2011).
39. L. Prieto del Val, J. L. Cantero, M. Atienza, APOE ε4 constrains engagement of encoding-related compensatory networks in amnestic mild cognitive impairment. *Hippocampus* **25**, 993–1007 (2015).
40. A. Bakker, G. L. Krauss, M. S. Albert, C. L. Speck, L. R. Jones, C. E. Stark, M. A. Yassa, S. S. Bassett, A. L. Shelton, M. Gallagher, Reduction of hippocampal hyperactivity improves cognition in amnestic mild cognitive impairment. *Neuron* **74**, 467–474 (2012).
41. J. J. Palop, L. Mucke, Network abnormalities and interneuron dysfunction in Alzheimer disease. *Nat. Rev. Neurosci.* **17**, 777–792 (2016).
42. Alzheimer's Association, 2015 Alzheimer's disease facts and figures. *Alzheimers Dement.* **11**, 332–384 (2015).
43. M. C. Hoenig, G. N. Bischof, Ö. A. Onur, J. Kukolja, F. Jessen, K. Fliessbach, B. Neumaier, G. R. Fink, E. Kalbe, A. Drzezga, T. van Eimeren; Alzheimer's Disease Neuroimaging Initiative, Level of education mitigates the impact of tau pathology on neuronal function. *Eur. J. Nucl. Med. Mol. Imaging* **46**, 1787–1795 (2019).

44. M. Bécu, D. Sheynikhovich, G. Tatur, C. P. Agathos, L. L. Bologna, J.-A. Sahel, A. Arleo, Age-related preference for geometric spatial cues during real-world navigation. *Nat. Hum. Behav.* **4**, 88–99 (2020).
45. H. Malkki, Alzheimer disease: Effects of the APOE ε4 allele on brain development. *Nat. Rev. Neurol.* **10**, 4 (2014).
46. A. L. Heffernan, C. Chidgey, P. Peng, C. L. Masters, B. R. Roberts, The neurobiology and age-related prevalence of the ε4 allele of apolipoprotein E in Alzheimer's disease cohorts. *J. Mol. Neurosci.* **60**, 316–324 (2016).
47. P. Berens, CircStat: A MATLAB toolbox for circular statistics. *J. Stat. Softw.* **31**, 1–21 (2009).
48. R Core Team, R: A Language and Environment for Statistical Computing (2018).
49. D. Bates, M. Mächler, B. M. Bolker, S. Walker, Fitting linear mixed-effects models using lme4. *J. Stat. Softw.* **67**, 1–48 (2014).
50. R. Lenth, emmeans: Estimated Marginal Means, aka Least-Squares Means (2019).
51. M. Hegarty, A. E. Richardson, D. R. Montello, K. Lovelace, I. Subbiah, Development of a self-report measure of environmental spatial ability. *Intelligence* **30**, 425–447 (2002).
52. Ø. Langsrud, ANOVA for unbalanced data: Use Type II instead of Type III sums of squares. *Stat. Comput.* **13**, 163–167 (2003).
53. B. Fischl, A. van der Kouwe, C. Destrieux, E. Halgren, F. Ségonne, D. H. Salat, E. Busa, L. J. Seidman, J. Goldstein, D. Kennedy, V. Caviness, N. Makris, B. Rosen, A. M. Dale, Automatically parcellating the human cerebral cortex. *Cereb. Cortex* **14**, 11–22 (2004).
54. R. S. Desikan, F. Ségonne, B. Fischl, B. T. Quinn, B. C. Dickerson, D. Blacker, R. L. Buckner, A. M. Dale, R P. Maguire, B. T. Hyman, M. S. Albert, R. J. Killiany, An automated labeling system for subdividing the human cerebral cortex on MRI scans into gyral based regions of interest. *Neuroimage* **31**, 968–980 (2006).
55. T. N. Tombaugh, N. J. McIntyre, The mini-mental state examination: A comprehensive review. *J. Am. Geriatr. Soc.* **40**, 922–935 (1992).
56. R. C. Petersen, G. E. Smith, S. C. Waring, R. J. Ivnik, E. G. Tangalos, E. Kokmen, Mild cognitive impairment: Clinical characterization and outcome. *Arch. Neurol.* **56**, 303–308 (1999).

57. M. S. Albert, S. T. DeKosky, D. Dickson, B. Dubois, H. H. Feldman, N. C. Fox, A. Gamst, D. M. Holtzman, W. J. Jagust, R. C. Petersen, P. J. Snyder, M. C. Carrillo, B. Thies, C. H. Phelps, The diagnosis of mild cognitive impairment due to Alzheimer's disease: Recommendations from the National Institute on Aging-Alzheimer's Association workgroups on diagnostic guidelines for Alzheimer's disease. *Alzheimers Dement.* **7**, 270–279 (2011).
58. C. A. Helmstaedter, M. Lendth, S. Lux, *Verbaler Lern- und Merkfähigkeitstest* (Beltz Test, 2001).
59. J. C. Morris, The clinical dementia rating (CDR): Current version and scoring rules. *Neurology* **43**, 2412–2414 (1993).
60. J. C. Morris, A. Heyman, R. C. Mohs, J. P. Hughes, G. van Belle, G. Fillenbaum, E. D. Mellits, C. Clark, The Consortium to Establish a Registry for Alzheimer's Disease (CERAD). Part I. Clinical and neuropsychological assessment of Alzheimer's disease. *Neurology* **39**, 1159–1165 (1989).
61. A. Coutrot, E. Manley, D. Yesiltepe, R. C. Dalton, J. M. Wiener, C. Hölscher, M. Hornberger, H. J. Spiers, Cities have a negative impact on navigation ability: Evidence from 38 countries. bioRxiv 2020.01.23.917211 [**Preprint**]. 24 January 2020. https://doi.org/10.1101/2020.01.23.917211.
62. D. Bush, N. Burgess, Advantages and detection of phase coding in the absence of rhythmicity. *Hippocampus* **30**, 745–762 (2020).
63. B. J. Kraus, M. P. Brandon, R. J Robinson II, M. A. Connerney, M. E. Hasselmo, H. Eichenbaum, During running in place, grid cells integrate elapsed time and distance run. *Neuron* **88**, 578–589 (2015).
